# Improvement in residual paravalvular leakage after transcatheter aortic valve replacement with a self-expanding valve: ACURATE neo2 versus ACURATE neo

**DOI:** 10.1007/s12928-025-01170-1

**Published:** 2025-07-20

**Authors:** Yoichi Sugiyama, Hirokazu Miyashita, Sebastian Dahlbacka, Tommi Vähäsilta, Tiina Vainikka, Mikko Jalanko, Juho Viikilä, Mika Laine, Noriaki Moriyama

**Affiliations:** 1 Department of Cardiology and Catheterization Laboratories, Shonan Kamakura General Hospital, Okamoto 1370-1, Kamakura City, Kanagawa 247-8533 Japan; 2 Department of Cardiology, Heart and Lung Center, Helsinki University and Helsinki University Central Hospital, Haartmaninkatu 4, 00290 Helsinki, Finland

**Keywords:** Aortic stenosis, Paravalvular leakage, Self-expanding valve, Transcatheter aortic valve replacement

## Abstract

The paravalvular leakage (PVL) incidence immediately after transcatheter aortic valve replacement (TAVR) is reportedly lower with ACURATE neo2 than with ACURATE neo. However, the difference in the subsequent PVL improvement between these valves and the improvement mechanism remain unclear. This study aimed to compare the incidence of and changes in the PVL between these valves and investigate the factors associated with PVL improvement. We included 403 patients who underwent TAVR with ACURATE neo2 (*n* = 134) or ACURATE neo (*n* = 269). The study endpoint was PVL improvement, defined as a reduction in the PVL grade from mild or higher at discharge to none/trace at 3 months. The PVL incidence of mild grade or higher was significantly lower with ACURATE neo2 than with ACURATE neo at 3 months (15.7% vs. 25.7%; *p* = 0.0234) but did not differ significantly at discharge (33.3% vs. 27.5%; *p* = 0.2291). A significant decrease in the PVL was observed only with ACURATE neo2 (*p* < 0.0001). Multivariate analysis demonstrated that ACURATE neo2 and calcium volume of the aortic leaflets were independent predictors of PVL improvement. The incidence of mild grade or higher PVL was lower with ACURATE neo2 than with ACURATE neo at 3 months due to the subsequent improvement. Patient selection focusing on leaflet calcification is important for preventing PVL because severe calcification impedes PVL improvement.

**Graphical abstract:**

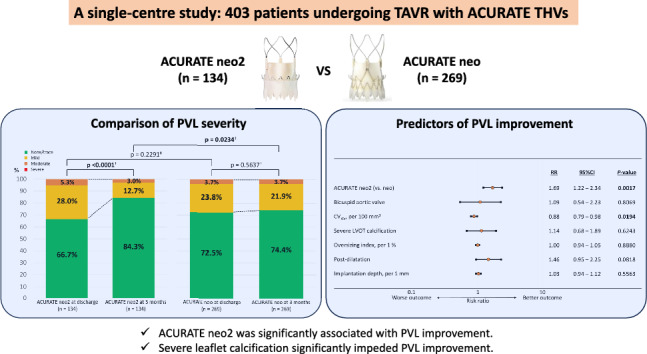

**Supplementary Information:**

The online version contains supplementary material available at 10.1007/s12928-025-01170-1.

## Introduction

Transcatheter aortic valve replacement (TAVR) is recognised as the established treatment for severe aortic stenosis (AS) across all levels of surgical risks [1–4]. Despite continuous procedural refinements, paravalvular leakage (PVL) remains a critical complication of TAVR in the life-long management of patients with AS because significant PVL, even mild, is associated with poor outcomes [5, 6]. ACURATE neo2 (Boston Scientifics, Marlborough, MA, USA), which has an improved outer skirt to mitigate PVL, received the CE-mark approval in 2021. According to several recent studies, it is associated with a lower incidence of moderate or severe grade PVL than ACURATE neo [7–9]. However, studies comparing the incidence of mild grade or higher PVL between the two valves are limited. Moreover, the difference in the subsequent changes in the PVL severity between these valves and the improvement mechanism of PVL remain unclear. Therefore, this study aimed to compare the incidence of mild grade or higher PVL at discharge and 3 months postoperatively and the change of PVL severity for 3 months between patients who underwent TAVR with ACURATE neo2 and ACURATE neo. Moreover, we investigated the factors associated with PVL improvement.

## Methods

### Study population

**Fig. 1:**
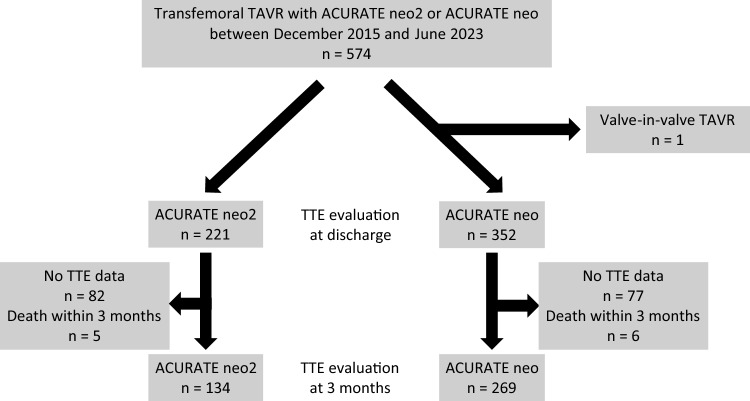
Study workflow. This study included patients who underwent TTE at discharge and the 3-month follow-up after TAVR. *TAVR* transcatheter aortic valve replacement, *TTE* transthoracic echocardiography

We analysed 574 consecutive patients who underwent transfemoral TAVR with ACURATE neo2 or ACURATE neo between December 2015 and June 2023 at Helsinki University Central Hospital. This study included 403 patients (ACURATE neo2 group, *n* = 134; ACURATE neo group, *n* = 269) who had transthoracic echocardiography (TTE) data available both at discharge and 3 months postoperatively. One patient who underwent valve-in-valve TAVR and 170 patients without the 3-month postoperative TTE data due to loss to follow-up or death were excluded (Fig. 1). The size of the transcatheter heart valve (THV) was determined based on the manufacturer’s instructions or modified sizing recommendations by Kim et al. [10]. Indications for TAVR, use of ACURATE THVs, and the approach site were decided by the cardiology team.

### Definitions and measurements

The presence of severe AS was defined based on the standard criteria. The operative risks of the patients were evaluated according to the Society of Thoracic Surgeons (STS) score [11, 12]. All multidetector computed tomography (CT) examinations were reviewed by three experienced interventional cardiologists (Y.S., H.M., and N.M.) using the 3mensio Structure Heart software (3mensio Medical Imaging BV, Bilthoven, The Netherlands). The annulus area and perimeter were measured by manually tracking the luminal contours in the double-oblique transverse plane. The calcium volume of the aortic leaflets (CV_AV_) was measured using contrast-enhanced multidetector CT with an 850-Hounsfield unit threshold for each leaflet [10, 13, 14]. The calcium severity of the left ventricular outflow tract (LVOT) was assessed using the semi-quantitative method described previously [15]. The oversizing index was calculated using the following formula: ((nominal valve perimeter − measured annular perimeter) / nominal valve perimeter) × 100 [16]. The implantation depth was measured from the fluoroscopy images using the institutional imaging software. The implantation depth was defined as the distance from the bottom of the non-coronary cusp to the ventricular end of the valve inflow strut in the final aortography during the TAVR procedure [17]. While the final view was a perpendicular deployment view, it could be adjusted according to the discretion of the operators. Pre- and post-TAVR TTE were performed by experienced echocardiography operators who were independent of the TAVR operators. PVL after TAVR was graded as none/trace, mild, moderate, or severe using qualitative or semi-quantitative methods according to the Valve Academic Research Consortium 3 (VARC-3) criteria [18]. Jet features assessed visually were used as the qualitative methods. As the semi-quantitative method, pressure half-time (PHT) with continuous wave Doppler was used. None/trace and mild PVL was defined as PHT > 500 ms; moderate was 200 ms ≤ PHT ≤ 500 ms; severe was PHT < 200 ms. Basically, PVL was graded visually by each echocardiographer. PHT was used as an auxiliary method. PVL improvement was defined as a change in the PVL severity in each patient from mild grade or higher at discharge to none/trace at 3 months postoperatively. Patients with mild grade or higher PVL at 3 months postoperatively were specified as them without PVL improvement. Patients with none/trace PVL at both time points were excluded from the analyses of PVL improvement. Besides, worsening of PVL was defined as an increase of at least one grade from discharge to 3 months. The clinical outcomes at 30 days were categorised using the VARC-2 criteria [19].

### Study endpoint

The endpoint was PVL improvement in each patient from discharge to 3-month follow-up. Multivariate analysis was performed to investigate the variables correlating with PVL improvement and mild grade or higher PVL at 3 months postoperatively.

### Statistical analysis

Data are presented as means ± standard deviations for normally distributed continuous variables or as medians and interquartile ranges (25–75%) for non-normally distributed continuous variables. Categorical variables are expressed as numeric values and percentages. Continuous variables were compared using the student’s *t*-test or Wilcoxon rank-sum test, depending on the variable distribution. Categorical variables were compared using the McNemar test for paired data and the chi-square statistics or Fisher exact test for unpaired data. Modified Poisson regression analyses were performed to investigate the significant variables for PVL improvement and mild grade or higher PVL at 3 months postoperatively. The multivariate regression models included pre-defined variables described as significant in previous reports, such as THV type, bicuspid aortic valve, CV_AV_, severe LVOT calcification, oversizing index, post-dilatation, and implantation depth [10, 20–22]. The CV_AV_ threshold for predicting mild grade or higher PVL at 3 months was evaluated by creating a receiver operating characteristic (ROC) curve. Subgroup analyses were performed based on aortic anatomical features (anatomy of native aortic valve and CV_AV_) and TTE data (maximum AV at baseline and PVL severity at discharge). The interaction analyses were performed for the aortic anatomical features, and *p*-values < 0.20 were considered significant. Kaplan–Meier analysis was performed using the log-rank test to compare 1-year composite outcome of all-cause death and/or heart failure rehospitalisation. Two-tailed *p*-values < 0.05 were considered statistically significant. All statistical analyses were performed using JMP, version 16 (SAS Institute, Cary, NC, USA) and EZR, version 1.64 (Jichi Medical University, Saitama, Japan).

## Results

### Baseline and procedural characteristics

This study evaluated 403 consecutive patients (ACURATE neo2 group, n = 134; ACURATE neo group, *n* = 269) (Fig. 1). Table 1 shows the baseline patient characteristics. The mean age was 81.0 ± 6.1 years, 72.0% of the participants were female, and the mean STS score was 4.0 ± 2.7%. The STS score was significantly lower in the ACURATE neo2 group than in the ACURATE neo group (3.5 ± 2.0% vs. 4.3 ± 3.0%; *p* = 0.0058). Other baseline variables did not differ significantly between the two groups. Table 2 shows the pre-procedural assessments and procedural data. Pre-procedural maximum aortic valve velocity (AV) was significantly lower in the ACURATE neo2 group than in the ACURATE neo group (4.0 ± 0.6 m/s vs. 4.2 ± 0.6 m/s; *p* = 0.0428). In the ACURATE neo2 group, the bicuspid aortic valve was less frequent (3.7% vs. 12.4%; *p* = 0.0060) and CV_AV_ was lower (195.3 [98.9–330.4] mm^3^ vs. 235.5 [132.8–383.1] mm^3^; *p* = 0.0255) compared to the ACURATE neo group. No significant differences were observed in the procedural variables between the groups. Comparison of baseline, pre-procedural, and procedural characteristics between the included patients with 3-month TTE data and the excluded patients without it are shown in **Online Resource** **1** and **Online Resource** **2** by each THV type. Baseline variables did not differ significantly except for prior coronary artery bypass graft in ACURATE neo. As for pre-procedural and procedural characteristics, maximum AV was significantly lower and implantation depth was significantly deeper in patients with 3-month TTE data than in those without it in ACURATE neo2. The rate of bicuspid aortic valve and CV_AV_ were not significantly different between patients with 3-month TTE data and those without it both in THV types.

**Table 1:** Baseline characteristics

	Total (*n* = 403)	ACURATE neo2 (*n* = 134)	ACURATE neo (*n* = 269)	*p* value
*Baseline clinical data*				
Age, *y*	81.0 ± 6.1	81.4 ± 6.1	80.8 ± 6.2	0.3744
Female, *n* (%)	290 (72.0)	100 (74.6)	190 (70.6)	0.4003
BMI, kg/m^2^	27.1 ± 5.4	27.5 ± 5.8	26.8 ± 5.1	0.2276
NYHA class ≥ III, *n* (%)	290 (72.0)	90 (67.2)	200 (74.4)	0.1303
STS score, %	4.0 ± 2.7	3.5 ± 2.0	4.3 ± 3.0	**0.0058**
Hypertension, *n* (%)	367 (91.1)	123 (91.8)	244 (90.7)	0.7191
Dyslipidaemia, *n* (%)	310 (76.9)	109 (81.3)	201 (74.7)	0.1372
Diabetes mellitus, *n* (%)	109 (27.1)	38 (28.4)	71 (26.4)	0.6758
Atrial fibrillation, *n* (%)	152 (37.7)	55 (41.0)	97 (36.1)	0.3307
Chronic kidney disease^a^, *n* (%)	172 (42.7)	51 (38.1)	121 (45.0)	0.1857
Haemodialysis, *n* (%)	2 (0.5)	1 (0.8)	1 (0.4)	1.0000
COPD, *n* (%)	85 (21.1)	24 (17.9)	61 (22.7)	0.2692
Peripheral artery disease, *n* (%)	40 (9.9)	12 (9.0)	28 (10.4)	0.6457
Prior PCI, *n* (%)	109 (27.1)	35 (26.1)	74 (27.5)	0.7673
Prior CABG, *n* (%)	16 (4.0)	6 (4.5)	10 (3.7)	0.7127
Prior stroke, *n* (%)	41 (10.2)	13 (9.7)	28 (10.4)	0.8248
Prior PPI, *n* (%)	38 (9.4)	13 (9.7)	25 (9.3)	0.8950
*Baseline electrocardiographic data*				
CRBBB, *n* (%)	30 (7.4)	11 (8.2)	19 (7.1)	0.6797
CLBBB, *n* (%)	29 (7.2)	11 (8.2)	18 (6.7)	0.5786

Values are means ± SD or %. *P*-values in **bold** are statistically significant. ^a^Estimated glomerular filtration rate < 60 ml/min/1.73m^2^

*BMI* body mass index; *CABG* coronary artery bypass graft, *CLBBB* complete left bundle branch block, *COPD* chronic obstructive pulmonary disease, *CRBBB* complete right bundle branch block, *NYHA* New York Heart Association, *PCI* percutaneous coronary intervention, *PPI* permanent pacemaker implantation, *SD* standard deviations, STS score = Society of Thoracic Surgeons score

**Table 2:** Pre-procedural assessment and procedural characteristics

	Total (*n* = 403)	ACURATE neo2 (*n* = 134)	ACURATE neo (*n* = 269)	*p* value
*Pre-procedural TTE data*				
LVEF, %	58.9 ± 11.1	59.9 ± 10.9	58.4 ± 11.2	0.2097
Mean APG, mmHg	42.1 ± 13.0	40.6 ± 12.1	42.8 ± 13.4	0.1088
Maximum AV, m/s	4.1 ± 0.6	4.0 ± 0.6	4.2 ± 0.6	**0.0428**
Aortic valve area, cm^2^	0.7 ± 0.2	0.7 ± 0.2	0.7 ± 0.2	0.0592
*Pre-procedural CT data*				
Annulus area, mm^3^	429.0 ± 59.6	426.7 ± 57.3	430.1 ± 60.8	0.5895
Annulus perimeter, mm	74.9 ± 5.2	74.6 ± 5.1	75.0 ± 5.3	0.4498
LVOT mean diameter, mm	23.1 ± 2.6	23.1 ± 2.1	23.1 ± 2.8	0.9895
STJ mean diameter, mm	27.4 ± 3.1	27.4 ± 3.1	27.4 ± 3.0	0.8949
SOV mean diameter, mm	30.2 ± 8.5	30.2 ± 2.8	30.1 ± 10.2	0.9032
Bicuspid aortic valve, *n* (%)	38 (9.5)	5 (3.7)	33 (12.4)	**0.0060**
CV_AV_, mm^3^	222.4 [129.5–380.5]	195.3 [98.9–330.4]	235.5 [132.8–383.1]	**0.0255**
LCC CV_AV_, mm^3^	51.0 [23.3–96.6]	45.1 [17.2–88.1]	50.8 [24.6–90.5]	0.0657
RCC CV_AV_, mm^3^	50.1 [22.5–102.1]	48.1 [14.5–88.1]	50.1 [24.8–106.8]	0.0965
NCC CV_AV_, mm^3^	102.7 [48.8–183.7]	74.3 [34.4–167.2]	108.9 [50.9–187.2]	0.0623
Severe LVOT calcification, *n* (%)	29 (7.3)	10 (7.5)	19 (7.2)	0.9152
*Procedural data*				
THV size, *n* (%)				0.1614
Small (23 mm)	93 (23.1)	31 (23.1)	62 (23.1)	
Medium (25 mm)	175 (43.4)	66 (49.3)	109 (40.5)	
Large (27 mm)	135 (33.5)	37 (27.6)	98 (36.4)	
Oversizing index, %	5.4 ± 3.7	5.3 ± 3.4	5.5 ± 3.9	0.6851
Pre-dilatation, *n* (%)	399 (99.0)	134 (100.0)	265 (98.5)	0.3063
Post-dilatation, *n* (%)	33 (8.2)	8 (6.0)	25 (9.3)	0.2516
Implantation depth, mm	4.1 ± 1.9	4.4 ± 2.3	4.0 ± 1.6	0.0519
Second valve implantation, *n* (%)	1 (0.3)	0 (0)	1 (0.4)	1.0000
Coronary artery obstruction, *n* (%)	0 (0)	0 (0)	0 (0)	1.0000
Annulus rupture, *n* (%)	0 (0)	0 (0)	0 (0)	1.0000

Values are means ± SD, %, or medians [interquartile ranges]. *P*-values in **bold** are statistically significant

*APG* aortic valve pressure gradient, *AV* aortic valve velocity, *CT* computed tomography, *CV*_*AV*_ calcium volume of the aortic leaflets, *LCC* left coronary cusp, *LVEF* left ventricular ejection fraction, *LVOT* left ventricular outflow tract, *NCC* non-coronary cusp, *RCC* right coronary cusp, *SD* standard deviations, *SOV* sinus of Valsalva, *STJ* sinotubular junction, *THV* transcatheter heart valve, *TTE* transthoracic echocardiography

### Haemodynamic outcomes at discharge and 3 months

**Fig. 2:**
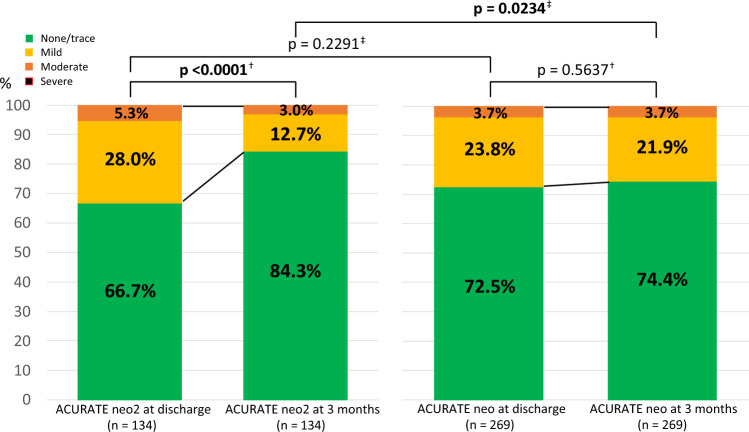
Comparison of mild grade or higher paravalvular leakage between the ACURATE neo2 and ACURATE neo groups at discharge and 3 months postoperatively. The ACURATE neo2 group shows a significant reduction in the incidence of mild grade or higher PVL at 3 months postoperatively compared to the ACURATE neo group. ^†^P-values show the results of the McNemar test for paired data comparing the incidence of mild grade or higher PVL between discharge and 3 months for each valve type. The ACURATE neo2 group shows a significantly lower incidence of mild grade or higher PVL than the ACURATE neo group at 3 months. ^‡^*P*-values show the results of the chi-square test for unpaired data comparing the incidence of mild grade or higher PVL between the two valve types at each time. *P*-values in bold are statistically significant. *PVL* paravalvular leakage

Table 3 shows the haemodynamic outcomes at discharge and 3 months after TAVR. The mean aortic valve pressure gradient (APG) and maximum AV were significantly higher in the ACURATE neo2 group than in the ACURATE neo group at discharge (mean APG: 10.0 ± 4.1 mmHg vs. 9.0 ± 5.1 mmHg, *p* = 0.0447; maximum AV: 2.1 ± 0.4 m/s vs. 1.9 ± 0.5 m/s, *p* = 0.0020). However, they did not differ significantly between the two groups at 3 months postoperatively (mean APG: 7.9 ± 3.8 mmHg vs. 7.6 ± 4.3 mmHg, *p* = 0.6124; maximum AV: 1.9 ± 0.4 m/s vs. 1.8 ± 0.5 m/s, *p* = 0.1279). On the other hand, the incidence of mild grade or higher PVL did not differ significantly between the two groups at discharge (33.3% vs. 27.5%; *p* = 0.2291) but was significantly lower in the ACURATE neo2 group than in the ACURATE neo group at 3 months (15.7% vs. 25.7%; *p* = 0.0234) on the chi-square statistics for unpaired data. The McNemar test for paired data revealed that the incidence of mild grade or higher PVL decreased significantly over time in the ACURATE neo2 group (33.3% at discharge vs. 15.7% at 3 months; *p* < 0.0001); however, it did not change significantly in the ACURATE neo group (27.5% at discharge vs. 25.7% at 3 months; *p* = 0.5637) (Fig. 2). PVL improvement in each patient was more frequent in the ACURATE neo2 group than in the ACURATE neo group (60.8% vs. 36.7%; *p* = 0.0043). Contrary, worsening of PVL was less frequent in the ACURATE neo2 group than in the ACURATE neo group (17.7% vs. 33.0%; *p* = 0.0438). **Online Resource** **3** shows a comparison of the change in PVL grades from discharge to 3 months between the ACURATE neo2 and ACURATE neo groups. **Online Resource** **4** shows the haemodynamic outcomes at discharge of the excluded patients without TTE data at 3 months postoperatively. The haemodynamic outcomes of the excluded patients were similar to those of the included patients. The proportion of different PVL grades at discharge was also similar between the included and excluded patients.

**Table 3:** Haemodynamic outcomes

	Total (*n* = 403)	ACURATE neo2 (*n* = 134)	ACURATE neo (*n* = 269)	*p* value
*TTE at discharge*				
LVEF, %	59.6 ± 10.8	61.1 ± 11.0	59.1 ± 10.7	0.4530
Mean APG, mmHg	9.3 ± 4.8	10.0 ± 4.1	9.0 ± 5.1	**0.0447**
Maximum AV, m/s	2.0 ± 0.5	2.1 ± 0.4	1.9 ± 0.5	**0.0020**
PVL ≥ mild, *n* (%)	118 (29.4)	44 (33.3)	74 (27.5)	0.2291
PVL ≥ moderate, *n* (%)	19 (4.7)	9 (6.8)	10 (3.7)	0.1696
*PVL*, *n* (%)				0.4533
Severe	0 (0)	0 (0)	0 (0)	
Moderate	17 (4.2)	7 (5.3)	10 (3.7)	
Mild	101 (25.2)	37 (28.0)	64 (23.8)	
None/trace	283 (70.6)	88 (66.7)	195 (72.5)	
TTE at 3 months				
LVEF, %	60.3 ± 9.7	59.6 ± 8.4	60.6 ± 10.1	0.4392
Mean APG, mmHg	7.7 ± 4.1	7.9 ± 3.8	7.6 ± 4.3	0.6124
Maximum AV, m/s	1.8 ± 0.4	1.9 ± 0.4	1.8 ± 0.5	0.1279
PVL ≥ mild, *n* (%)	90 (22.3)	21 (15.7)	69 (25.7)	**0.0234**
PVL ≥ moderate, *n* (%)	14 (3.5)	4 (3.0)	10 (3.7)	1.0000
*PVL*, *n* (%)				0.0693
Severe	0 (0)	0 (0)	0 (0)	
Moderate	14 (3.5)	4 (3.0)	10 (3.7)	
Mild	76 (18.9)	17 (12.7)	59 (21.9)	
None/trace	313 (77.7)	113 (84.3)	200 (74.4)	
Improvement of PVL, *n* (%)	71/160 (44.4)	31/51 (60.8)	40/109 (36.7)	**0.0043**
Worsening of PVL, *n* (%)	45/160 (28.1)	9/51 (17.7)	36/109 (33.0)	**0.0438**

Values are means ± SD or %. *P*-values in **bold** are statistically significant

*APG* aortic valve pressure gradient, *AV* aortic valve velocity, *LVEF* left ventricular ejection fraction, *PVL* paravalvular leakage, *SD* standard deviations, *TTE* transthoracic echocardiography

### Independent predictors of PVL improvement and mild grade or higher PVL at 3 months

**Fig. 3:**
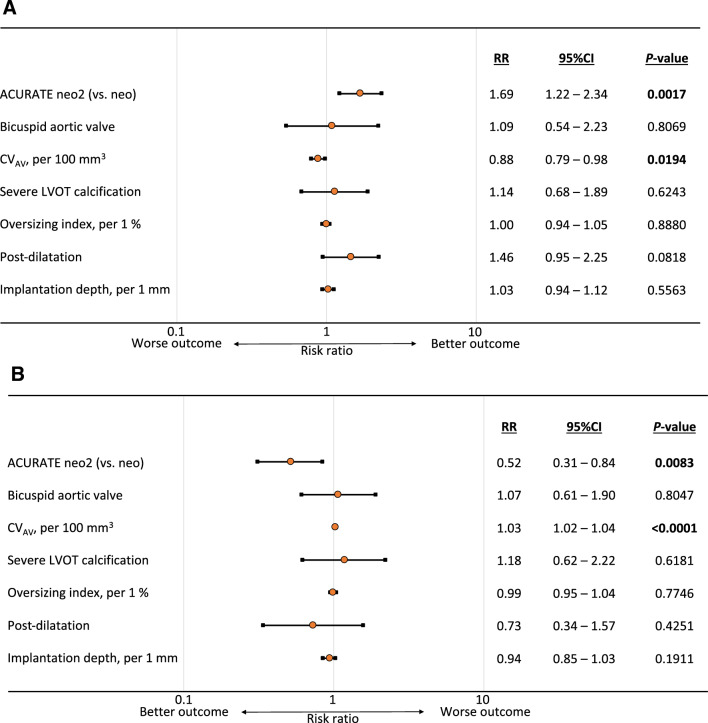
Multivariate analysis investigating the factors associated with paravalvular leakage improvement and mild grade or higher paravalvular leakage at 3 months postoperatively. **a** Use of ACURATE neo2 shows an independent association with PVL improvement, and CV_AV_ significantly impedes PVL improvement. **b** Use of ACURATE neo2 significantly prevents mild grade or higher PVL at 3 months postoperatively. CV_AV_ is independently associated with mild grade or higher PVL at 3 months postoperatively. *P*-values in bold are statistically significant. *CI* confidence interval, *CV*_*AV*_ calcium volume of the aortic leaflets, *LVOT* left ventricular outflow tract, *PVL* paravalvular leakage, *RR* risk ratio

**Fig. 4:**
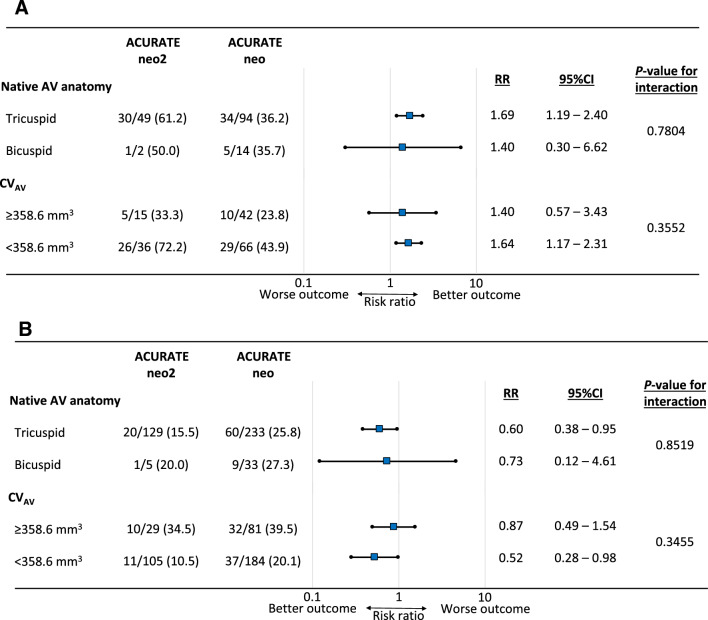
Subgroup analysis for paravalvular leakage improvement and mild grade or higher paravalvular leakage at 3 months postoperatively. **a** Use of ACURATE neo2 was associated with PVL improvement. There were no significant interactions between THV type and anatomy of native aortic valve and between THV type and CV_AV_. **b** Use of ACURATE neo2 prevented mild grade or higher PVL at 3 months postoperatively. There were no significant interactions between THV type and anatomy of native aortic valve and between THV type and CV_AV_. *AV* aortic valve, *CI* confidence interval, *CV*_*AV*_ calcium volume of the aortic leaflets, *PVL* paravalvular leakage, *RR* risk ratio, *THV* transcatheter heart valve

Modified Poisson regression analysis demonstrated that the use of ACURATE neo2 was independently associated with PVL improvement (risk ratio [RR]: 1.69; 95% confidence interval [CI] 1.22–2.34). On the other hand, CV_AV_ significantly impeded PVL improvement (RR: 0.88 per 100 mm^3^; 95% CI 0.79–0.98) (Fig. 3a). Moreover, ACURATE neo2 (RR: 0.52; 95% CI 0.31–0.84) and CV_AV_ (RR: 1.03 per 100 mm^3^; 95% CI 1.02–1.04) were significant predictors of mild grade or higher PVL at 3 months (Fig. 3b). **Online Resource** **5** shows the ROC curve evaluating the CV_AV_ threshold for predicting mild grade or higher PVL at 3 months. The CV_AV_ cut-off value was 358.6 mm^3^ with a sensitivity of 46.7% and specificity of 78.0% (area under the curve = 0.66; 95% CI 0.59–0.72). The CV_AV_ cut-off values in each valve are shown in **Online Resource** **6**. Based on aortic anatomical and TTE variables, the subgroup analyses confirmed the consistent effect of ACURATE neo2 in comparison with ACURATE neo for PVL improvement and none/trace PVL at 3 months (**Online Resource** **7**–**Online Resource** **10**). There were no significant interactions between THV type and anatomy of native aortic valve and between THV type and CV_AV_ (Fig. 4).

### Clinical outcomes

Table 4 shows the clinical outcomes at 30 days. No significant differences in any of the clinical outcomes were observed between the two groups. **Online Resource** **11** reveals the comparison of all-cause death and/or heart failure rehospitalisation between patients with improvement of PVL and those without. No significant difference in the composite outcome was detected at 1 year.

**Table 4:** Clinical outcomes at 30 days

	Total (*n* = 403)	ACURATE neo2 (*n* = 134)	ACURATE neo (*n* = 269)	*p* value
All-cause mortality, *n* (%)	0 (0)	0 (0)	0 (0)	1.0000
Major vascular complications, *n* (%)	35 (8.7)	11 (8.2)	24 (8.9)	0.8108
Life-threatening or major bleeding, *n* (%)	44 (10.9)	12 (9.0)	32 (11.9)	0.3725
Acute kidney injury, *n* (%)	5 (1.2)	2 (1.5)	3 (1.1)	1.0000
Stroke/TIA, *n* (%)	19 (4.7)	6 (4.5)	13 (4.8)	0.8741
PPI, *n* (%)	16/365 (4.4)	7/121 (5.8)	9/244 (3.7)	0.3570

Values are %

*PPI* permanent pacemaker implantation, *TIA* transient ischemic attack

## Discussion

We performed a retrospective comparison of the PVL incidence at hospital discharge and the 3-month follow-up after TAVR and the change of PVL severity from discharge to 3 months between ACURATE neo2 and ACURATE neo. The major findings of this investigation were as follows: (1) the incidence of mild grade or higher PVL was significantly lower with ACURATE neo2 than with ACURATE neo at 3 months but did not differ significantly at discharge; (2) the incidence of mild grade or higher PVL decreased significantly over time only with ACURATE neo2 use; (3) ACURATE neo2 was independently associated with PVL improvement and prevented mild grade or higher PVL at 3 months; and (4) CV_AV_ correlated negatively with PVL improvement and resulted in mild grade or higher PVL at 3 months.

### Impact of mild grade or higher PVL on long-term mortality after TAVR

ACURATE neo is a first-generation device with a self-expandable nitinol stent frame and supra-annular bovine leaflets. While ACURATE neo demonstrated favourable haemodynamic and clinical outcomes in several single-arm studies [23, 24], two randomised controlled trials revealed that ACURATE neo was significantly associated with a higher incidence of moderate or severe PVL than Sapien 3 (Edwards Lifesciences, Irvine, CA, USA) and Evolut R/PRO (Medtronic Inc., Minneapolis, MN, USA) [25, 26]. Thus, ACURATE neo2 was developed as a new device with a taller outer sealing skirt to overcome this issue. Consequently, ACURATE neo2 significantly reduced the incidence of moderate or severe PVL compared to ACURATE neo [7–9]. However, studies comparing the incidence of mild grade or higher PVL between these two valves are limited. Because recent studies have reported that even mild PVL is associated with an increased risk of 5-year mortality [5, 6], it is critical to minimise the incidence of PVL, regardless of the severity, in the era of well-established TAVR expanding to low-risk populations with a long life expectancy.

### Incidence of mild grade or higher PVL in the new generation THV

The incidence of mild grade or higher PVL with ACURATE neo2 in previous reports was 32.5–50.0% at discharge (mild: 30.8–46.5%; moderate/severe: 1.7–3.5%) and 48.5% at 3 months (mild: 45.0%; moderate/severe: 3.5%) [7–9]. In our study, the incidence of moderate or severe PVL at discharge was higher than that reported previously, which could be attributed to the lower rate of post-dilatation in this study than in the previous studies (6.0% vs. 31.7–43.3%) [7–9]. However, the incidence of moderate or severe PVL at 3 months was comparable and the incidence of mild PVL at 3 months was lower in this study than in the previous study. This could be explained by the patient selection in terms of the severity and distribution of leaflet calcification. At our institution, other valves tend to be selected for patients with high amounts of leaflet calcification and/or extremely asymmetric leaflet calcifications. This patient selection may have improved during the periods from ACURATE neo use to ACURATE neo2 use at our institution. Notably, CV_AV_ was significantly lower in the ACURATE neo2 group than in the ACURATE neo group. When leaflet calcification is not severe, ACURATE neo2 can further expand after valve deployment, even with its low radial force. Thus, ACURATE neo2 can further contact with the surrounding tissue, which leads to PVL improvement. Owing to this patient selection, the incidence of mild grade or higher PVL at 3 months was lower than that reported previously, despite the lower rate of post-dilatation in our study. Besides, we tended to accept PVL immediately after valve deployment more frequently in the era of ACURATE neo2 than that of ACURATE neo. In the ACURATE neo2 era, we anticipated that PVL immediately after valve deployment could be improved over time due to further expansion of the stent frame with the improved patient selection and further contact with the improved outer skirt. Therefore, the rate of post-dilatation was lower in the era of ACURATE neo2 than that of ACURATE neo. That could result in the insignificant difference in the incidence of mild grade or higher PVL at discharge between the two valves.

### Impact of the THV generation on PVL

The significant reduction in the PVL severity with ACURATE neo2 was consistent with the findings of a previous report [27]. In contrast, the results with ACURATE neo contradicted those of another study [28]. Kim et al. described that ACURATE neo showed PVL improvement at 12 months rather than at 1 month after TAVR. This discrepancy might imply that tissue ingrowth covering the paravalvular spaces occurred earlier with ACURATE neo2 due to the taller outer skirt and an active sealing mechanism, and the tissue ingrowth with ACURATE neo might not be sufficient to reduce the PVL at 3 months. In this study, the haemodynamic outcomes at 1 year could not be analysed because of the large amount of missing data. If the data were available, ACURATE neo might have shown a similar improvement. Nevertheless, the earlier improvement in the abnormal blood flow with ACURATE neo2 could be an advantage over ACURATE neo in minimising the left ventricular volume overload and damage to red blood cells that causes haemolysis.

### Impact of CVAV on PVL

ACURATE neo2 has been reported as a promising device for severe leaflet calcification [7]. However, CV_AV_ disturbed the PVL improvement in this study and resulted in mild grade or higher PVL at 3 months, regardless of the THV type, oversizing index, and post-dilatation rate. Therefore, patient selection based on the leaflet calcification severity is important for preventing PVL, even when using ACURATE neo2. The CV_AV_ cut-off value may be valuable for THV selection.

### PVL improvement mechanism with ACURATE THVs

Our results support the hypothesis that tissue ingrowth promoted by contact between the skirt of the THV and surrounding tissues is the most important factor for PVL improvement with ACURATE THVs. Compared to ACURATE neo, the taller outer skirt of ACURATE neo2 could enhance the contact area, and the active sealing mechanism could increase the contact time. Therefore, tissue ingrowth would be enhanced more strongly with ACURATE neo2 than with ACURATE neo, which might contribute to PVL improvement. Furthermore, an effect of further expansion of the stent frame as a PVL improvement mechanism might be expected only in patients without severe leaflet calcification. We hypothesised that severe leaflet calcification might disturb PVL improvement by impeding contact with the surrounding tissues and further expansion of the stent frame. ACURATE THVs, whose radial forces are lower than those of Medtronic self-expanding valves [29, 30], may show a lower CV_AV_ threshold for predicting significant PVL. Therefore, careful patient selection focusing on leaflet calcification should be performed. In contrast, the incidence of mild grade or higher PVL with ACURATE neo2 could be comparable to that of the newest Medtronic self-expanding valve when patients are selected appropriately [31]. The improvement of that patient selection during the periods from ACURATE neo use to ACURATE neo2 use could result in the more frequent improvement of PVL in the ACURATE neo2 group than the ACURATE neo group. Moreover, our study showed that unnecessary post-dilatation could be avoided to reduce the incidence of permanent pacemaker implantation in patients without severe leaflet calcification because the incidence of mild grade or higher PVL at 3 months with ACURATE neo2 was low despite the low post-dilatation rate.

### Study limitations

This study has several limitations. First, this was a retrospective, observational study. The TAVR indications and THV selection depended on institutional discretion. Second, our study lacked a core laboratory to analyse the TTE, CT, and fluoroscopic imaging findings. Inter- and intra-observer variability may have affected our results. Especially, PVL severity was basically evaluated with the qualitative methods of TTE. The use of the semi-quantitative method was auxiliary and depended on the discretion of each echocardiographer. Third, the improvement in the learning curves of the implantation techniques during the periods from ACURATE neo use to ACURATE neo2 use might have influenced the study endpoint. Fourth, the post-dilatation rate was quite low in this study. If post-dilatation had been performed more often, the incidence of mild grade or higher PVL at discharge would have decreased, and the subsequent PVL improvement with the ACURATE neo2 might not have been significant. Fifth, PVL was evaluated only at 3-month follow-up and could not be analysed at 1-month, 6-month, and 1-year follow-ups because of the large amount of missing data. Therefore, the longitudinal change of PVL could not be evaluated in more detail. Finally, many patients were excluded from our analyses because of missing 3-month postoperative TTE data. This could have produced biases in the analyses of PVL severity at 3 months and PVL improvement. Particularly, if patients showing mild grade or higher PVL at discharge in the ACURATE neo2 group were disproportionately excluded because of missing TTE data at 3 months, the reduction in the mild grade or higher PVL at 3 months could be attributed to the exclusion. However, no major difference was observed in the PVL severity at discharge between patients with 3-month TTE data and those without it in the ACURATE neo2 and ACURATE neo groups. Moreover, no major differences were detected in baseline, pre-procedural, and procedural characteristics between patients with 3-month TTE data and those without it.

## Conclusions

ACURATE neo2 showed significant improvement in PVL over time and a lower incidence of mild grade or higher PVL compared to ACURATE neo 3 months after TAVR. In contrast, severe leaflet calcification significantly impeded PVL improvement and resulted in mild grade or higher PVL at 3 months postoperatively. Severe leaflet calcification could result in less contact between the skirt of the THV and the surrounding tissue and impede further expansion of the stent frame, which leads to significant PVL. Therefore, patient selection considering aortic leaflet calcification is crucial, even when using ACURATE neo2.

## Supplementary Information

Below is the link to the electronic supplementary material.

Supplementary file1 (DOCX 34 KB)

Supplementary file2 (PPTX 224 KB)

## Data Availability

The data that support the findings of this study are available from the corresponding author upon reasonable request.
