# Supplementary material for: Improvement in residual paravalvular leakage after transcatheter aortic valve replacement with a self-expanding valve: ACURATE neo2 versus ACURATE neo
Source: Cardiovasc Interv Ther. 2025 Jul 20;40(4):909–20. doi: 10.1007/s12928-025-01170-1 (PMC12431930; doi:10.1007/s12928-025-01170-1)
Supplement: Supplementary file 1 — Supplementary file1 (DOCX 34 KB) [file 12928_2025_1170_MOESM1_ESM.docx]

**Online Resource 1. Comparison of baseline characteristics between patients with 3-month TTE data and those without it in each valve**

|  | ACURATE neo2 | | | ACURATE neo | | |
| --- | --- | --- | --- | --- | --- | --- |
|  | With (n = 134) | Without (n = 87) | p value | With (n = 269) | Without (n = 83) | p value |
| Baseline clinical data |  |  |  |  |  |  |
| Age, y | 81.4 ± 6.1 | 81.9 ± 5.4 | 0.4977 | 80.8 ± 6.2 | 82.0 ± 6.2 | 0.1225 |
| Female, n (%) | 100 (74.6) | 72 (82.8) | 0.1551 | 190 (70.6) | 57 (68.7) | 0.7333 |
| BMI, kg/m^2^ | 27.5 ± 5.8 | 26.6 ± 7.3 | 0.3065 | 26.8 ± 5.1 | 27.4 ± 4.9 | 0.3990 |
| NYHA class ≥Ⅲ, n (%) | 90 (67.2) | 58 (66.7) | 0.9388 | 200 (74.4) | 61 (73.5) | 0.8763 |
| STS score, % | 3.5 ± 2.0 | 3.3 ± 1.8 | 0.6242 | 4.3 ± 3.0 | 4.3 ± 2.4 | 0.9820 |
| Hypertension, n (%) | 123 (91.8) | 78 (89.7) | 0.5887 | 244 (90.7) | 73 (88.0) | 0.4635 |
| Dyslipidaemia, n (%) | 109 (81.3) | 74 (85.1) | 0.4747 | 201 (74.7) | 57 (68.7) | 0.2764 |
| Diabetes mellitus, n (%) | 38 (28.4) | 25 (28.7) | 0.9516 | 71 (26.4) | 21 (25.3) | 0.8430 |
| Atrial fibrillation, n (%) | 55 (41.0) | 32 (36.8) | 0.5262 | 97 (36.1) | 36 (43.4) | 0.2296 |
| Chronic kidney disease^a^, n (%) | 51 (38.1) | 39 (44.8) | 0.3171 | 121 (45.0) | 35 (42.2) | 0.6520 |
| Haemodialysis, n (%) | 1 (0.8) | 0 (0) | 1.0000 | 1 (0.4) | 1 (1.2) | 0.4165 |
| COPD, n (%) | 24 (17.9) | 21 (24.1) | 0.2614 | 61 (22.7) | 22 (26.5) | 0.4725 |
| Peripheral artery disease, n (%) | 12 (9.0) | 5 (5.8) | 0.4476 | 28 (10.4) | 11 (13.3) | 0.4705 |
| Prior PCI, n (%) | 35 (26.1) | 18 (20.7) | 0.3557 | 74 (27.5) | 25 (30.1) | 0.6437 |
| Prior CABG, n (%) | 6 (4.5) | 3 (3.5) | 1.0000 | 10 (3.7) | 9 (10.8) | **0.0120** |
| Prior stroke, n (%) | 13 (9.7) | 8 (9.2) | 0.9002 | 28 (10.4) | 10 (12.1) | 0.6740 |
| Prior PPI, n (%) | 13 (9.7) | 8 (9.2) | 0.9002 | 25 (9.3) | 6 (7.2) | 0.5617 |
| Baseline electrocardiographic data |  |  |  |  |  |  |
| CRBBB, n (%) | 11 (8.2) | 7 (8.3) | 0.9741 | 19 (7.1) | 8 (9.6) | 0.4409 |
| CLBBB, n (%) | 11 (8.2) | 9 (10.7) | 0.5329 | 18 (6.7) | 6 (7.2) | 0.8651 |

Values are means ± SD or %. P-values in **bold** are statistically significant. ^a^Estimated glomerular filtration rate <60 ml/min/1.73m^2^.

BMI = body mass index; CABG = coronary artery bypass graft; CLBBB = complete left bundle branch block; COPD = chronic obstructive pulmonary disease; CRBBB = complete right bundle branch block; NYHA = New York Heart Association; PCI = percutaneous coronary intervention; PPI = permanent pacemaker implantation; SD = standard deviations; STS score = Society of Thoracic Surgeons score.

**Online Resource 2. Comparison of pre-procedural and procedural characteristics between patients with 3-month TTE data and those without it in each valve**

|  | ACURATE neo2 | | | ACURATE neo | | |
| --- | --- | --- | --- | --- | --- | --- |
|  | With (n = 134) | Without (n = 87) | p value | With (n = 269) | Without (n = 83) | p value |
| Pre-procedural TTE data |  |  |  |  |  |  |
| LVEF, % | 59.9 ± 10.9 | 58.9 ± 10.3 | 0.4953 | 58.4 ± 11.2 | 61.0 ± 10.5 | 0.0607 |
| Mean APG, mmHg | 40.6 ± 12.1 | 42.7 ± 8.3 | 0.1514 | 42.8 ± 13.4 | 43.1 ± 12.2 | 0.8438 |
| Maximum AV, m/s | 4.0 ± 0.6 | 4.2 ± 0.4 | **0.0297** | 4.2 ± 0.6 | 4.2 ± 0.6 | 0.9809 |
| Aortic valve area, cm^2^ | 0.7 ± 0.2 | 0.7 ± 0.2 | 0.0952 | 0.7 ± 0.2 | 0.7 ± 0.2 | 0.2259 |
| Pre-procedural CT data |  |  |  |  |  |  |
| Annulus area, mm^3^ | 426.7 ± 57.3 | 422.4 ± 51.3 | 0.5714 | 430.1 ± 60.8 | 430.6 ± 57.1 | 0.9488 |
| Annulus perimeter, mm | 74.6 ± 5.1 | 74.1 ± 4.7 | 0.4363 | 75.0 ± 5.3 | 75.1 ± 4.9 | 0.9488 |
| LVOT mean diameter, mm | 23.1 ± 2.1 | 23.1 ± 2.0 | 0.9672 | 23.1 ± 2.8 | 23.1 ± 2.1 | 0.9825 |
| STJ mean diameter, mm | 27.4 ± 3.1 | 27.3 ± 3.0 | 0.7064 | 27.4 ± 3.0 | 27.8 ± 2.9 | 0.2374 |
| SOV mean diameter, mm | 30.2 ± 2.8 | 30.2 ± 2.5 | 0.8665 | 30.1 ± 10.2 | 29.7 ± 4.9 | 0.7294 |
| Bicuspid aortic valve, n (%) | 5 (3.7) | 3 (3.5) | 1.0000 | 33 (12.4) | 9 (11.0) | 0.7281 |
| CV_AV_, mm^3^ | 195.3 [98.9–330.4] | 225.2 [121.2–442.6] | 0.0532 | 235.5 [132.8–383.1] | 216.5 [146.0–381.0] | 0.9784 |
| LCC CV_AV_, mm^3^ | 45.1 [17.2–88.1] | 63.8 [23.7–107.7] | **0.0203** | 50.8 [24.6–90.5] | 56.5 [27.7–113.1] | 0.3187 |
| RCC CV_AV_, mm^3^ | 48.1 [14.5–88.1] | 58.9 [22.1–120.1] | 0.1186 | 50.1 [24.8–106.8] | 50.5 [23.8–94.1] | 0.8818 |
| NCC CV_AV_, mm^3^ | 74.3 [34.4–167.2] | 109.4 [54.7–195.7] | 0.0856 | 108.9 [50.9–187.2] | 95.5 [57.5–159.5] | 0.5837 |
| Severe LVOT calcification, n (%) | 10 (7.5) | 4 (4.6) | 0.5735 | 19 (7.2) | 7 (8.5) | 0.6812 |
| Procedural data |  |  |  |  |  |  |
| THV size, n (%) |  |  | 0.4997 |  |  | 0.9535 |
| Small (23 mm) | 31 (23.1) | 23 (26.4) |  | 62 (23.1) | 18 (21.7) |  |
| Medium (25 mm) | 66 (49.3) | 46 (52.9) |  | 109 (40.5) | 35 (42.2) |  |
| Large (27 mm) | 37 (27.6) | 18 (20.7) |  | 98 (36.4) | 30 (36.1) |  |
| Oversizing index, % | 5.3 ± 3.4 | 5.2 ± 3.0 | 0.8077 | 5.5 ± 3.9 | 5.6 ± 3.4 | 0.8812 |
| Pre-dilatation, n (%) | 134 (100.0) | 87 (100.0) | 1.0000 | 265 (98.5) | 81 (98.8) | 1.0000 |
| Post-dilatation, n (%) | 8 (6.0) | 9 (10.5) | 0.2231 | 25 (9.3) | 10 (12.2) | 0.4427 |
| Implantation depth, mm | 4.4 ± 2.3 | 3.8 ± 1.7 | **0.0346** | 4.0 ± 1.6 | 4.2 ± 2.1 | 0.3969 |
| Second valve implantation, n (%) | 0 (0) | 1 (1.2) | 0.3937 | 1 (0.4) | 0 (0) | 1.0000 |
| Coronary artery obstruction, n (%) | 0 (0) | 0 (0) | 1.0000 | 0 (0) | 0 (0) | 1.0000 |
| Annulus rupture, n (%) | 0 (0) | 0 (0) | 1.0000 | 0 (0) | 0 (0) | 1.0000 |

Values are means ± SD, %, or medians [interquartile ranges]. P-values in **bold** are statistically significant.

APG = aortic valve pressure gradient; AV = aortic valve velocity; CT = computed tomography; CV_AV_ = calcium volume of the aortic leaflets; LCC = left coronary cusp; LVEF = left ventricular ejection fraction; LVOT = left ventricular outflow tract; NCC = non-coronary cusp; RCC = right coronary cusp; SD = standard deviations; SOV = sinus of Valsalva; STJ = sinotubular junction; THV = transcatheter heart valve; TTE = transthoracic echocardiography.

**Online Resource 4. Haemodynamic outcomes at discharge of patients without transthoracic echocardiography data at 3 months**

|  | Total (n = 170) | ACURATE neo2 (n = 87) | ACURATE neo (n = 83) | p value |
| --- | --- | --- | --- | --- |
| TTE at discharge |  |  |  |  |
| LVEF, % | 57.9 ± 10.6 | 55.4 ± 10.8 | 60.3 ± 10.3 | 0.1779 |
| Mean APG, mmHg | 9.6 ± 4.2 | 10.3 ± 4.6 | 8.9 ± 3.7 | **0.0336** |
| Maximum AV, m/s | 2.1 ± 0.4 | 2.1 ± 0.5 | 2.0 ± 0.4 | **0.0308** |
| PVL ≥mild, n (%) | 47 (28.3) | 26 (30.6) | 21 (25.9) | 0.5051 |
| PVL ≥moderate, n (%) | 9 (5.4) | 3 (3.5) | 6 (7.4) | 0.3204 |
| PVL, n (%) |  |  |  | 0.2908 |
| Severe | 2 (1.2) | 0 (0) | 2 (2.5) |  |
| Moderate | 7 (4.2) | 3 (3.5) | 4 (4.9) |  |
| Mild | 38 (22.9) | 23 (27.1) | 15 (18.5) |  |
| None/trace | 119 (71.7) | 59 (69.4) | 60 (74.1) |  |

Values are means ± SD or %. P-values in **bold** are statistically significant.

APG = aortic valve pressure gradient; AV = aortic valve velocity; LVEF = left ventricular ejection fraction; PVL = paravalvular leakage; SD = standard deviations; TTE = transthoracic echocardiography.
