# Supplementary material for: Improvement in residual paravalvular leakage after transcatheter aortic valve replacement with a self-expanding valve: ACURATE neo2 versus ACURATE neo
Source: Cardiovasc Interv Ther. 2025 Jul 20;40(4):909–20. doi: 10.1007/s12928-025-01170-1 (PMC12431930; doi:10.1007/s12928-025-01170-1)
Supplement: Supplementary file 2 — Supplementary file2 (PPTX 224 KB) [file 12928_2025_1170_MOESM2_ESM.pptx]

## Slide 1
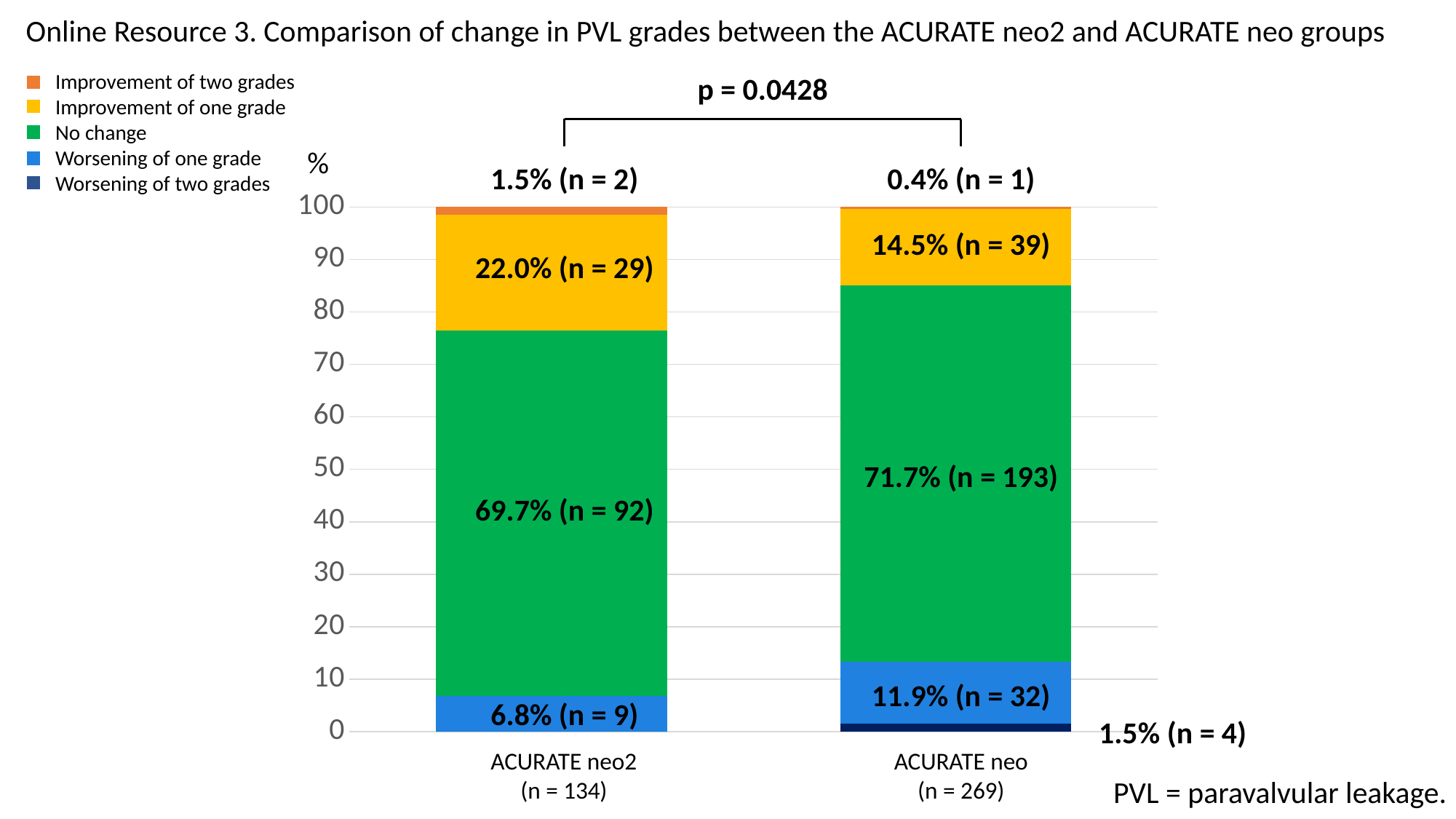

Online Resource 3. Comparison of change in PVL grades between the ACURATE neo2 and ACURATE neo groups
Improvement of two grades
Improvement of one grade
No change
Worsening of one grade
Worsening of two grades
p = 0.0428
%
1.5% (n = 2)
0.4% (n = 1)
### Chart
| Category | | | | | |
|---|---|---|---|---|---|
| Neo2 | 0.0 | 6.8 | 69.7 | 22.0 | 1.5 |
| Neo | 1.5 | 11.9 | 71.7 | 14.5 | 0.4 |14.5% (n = 39)
22.0% (n = 29)
71.7% (n = 193)
69.7% (n = 92)
11.9% (n = 32)
6.8% (n = 9)
1.5% (n = 4)
ACURATE neo2
(n = 134)
ACURATE neo
(n = 269)
PVL = paravalvular leakage.

## Slide 2
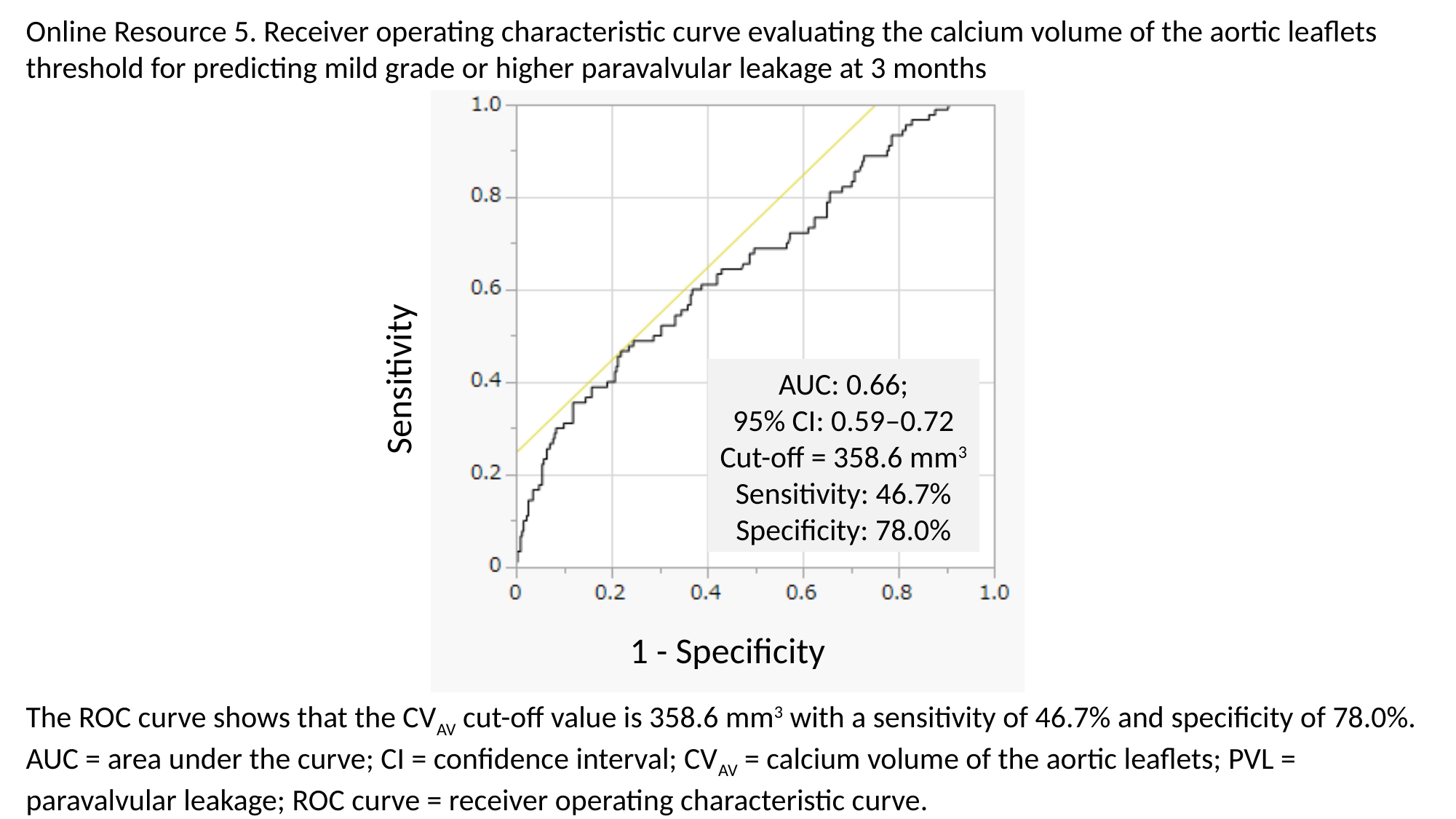

Online Resource 5. Receiver operating characteristic curve evaluating the calcium volume of the aortic leaflets threshold for predicting mild grade or higher paravalvular leakage at 3 months
Sensitivity
AUC: 0.66;
95% CI: 0.59–0.72
Cut-off = 358.6 mm3
Sensitivity: 46.7%
Specificity: 78.0%
1 - Specificity
The ROC curve shows that the CVAV cut-off value is 358.6 mm3 with a sensitivity of 46.7% and specificity of 78.0%.
AUC = area under the curve; CI = confidence interval; CVAV = calcium volume of the aortic leaflets; PVL = paravalvular leakage; ROC curve = receiver operating characteristic curve.

## Slide 3
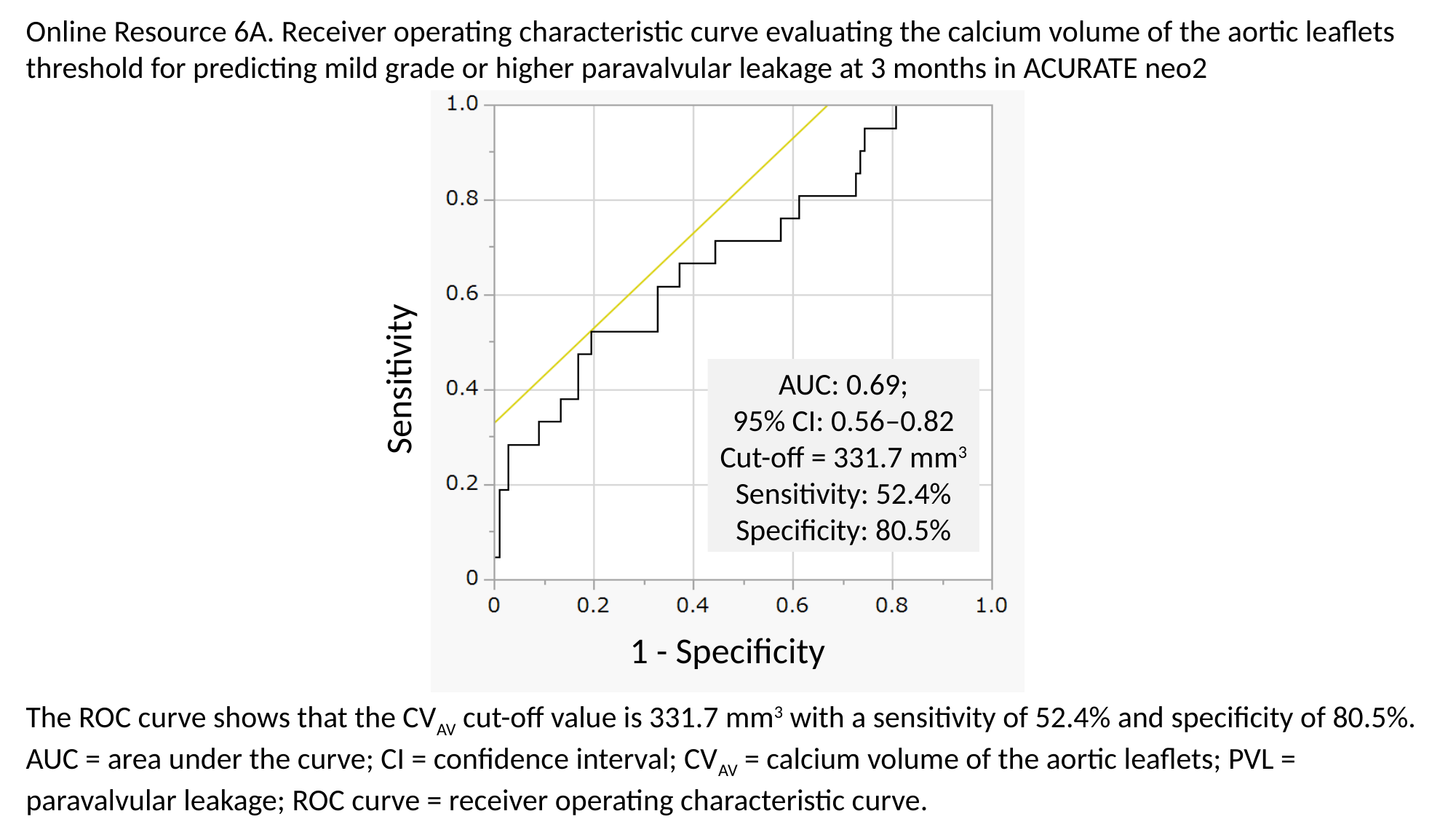

Online Resource 6A. Receiver operating characteristic curve evaluating the calcium volume of the aortic leaflets threshold for predicting mild grade or higher paravalvular leakage at 3 months in ACURATE neo2
Sensitivity
AUC: 0.69;
95% CI: 0.56–0.82
Cut-off = 331.7 mm3
Sensitivity: 52.4%
Specificity: 80.5%
1 - Specificity
The ROC curve shows that the CVAV cut-off value is 331.7 mm3 with a sensitivity of 52.4% and specificity of 80.5%.
AUC = area under the curve; CI = confidence interval; CVAV = calcium volume of the aortic leaflets; PVL = paravalvular leakage; ROC curve = receiver operating characteristic curve.

## Slide 4
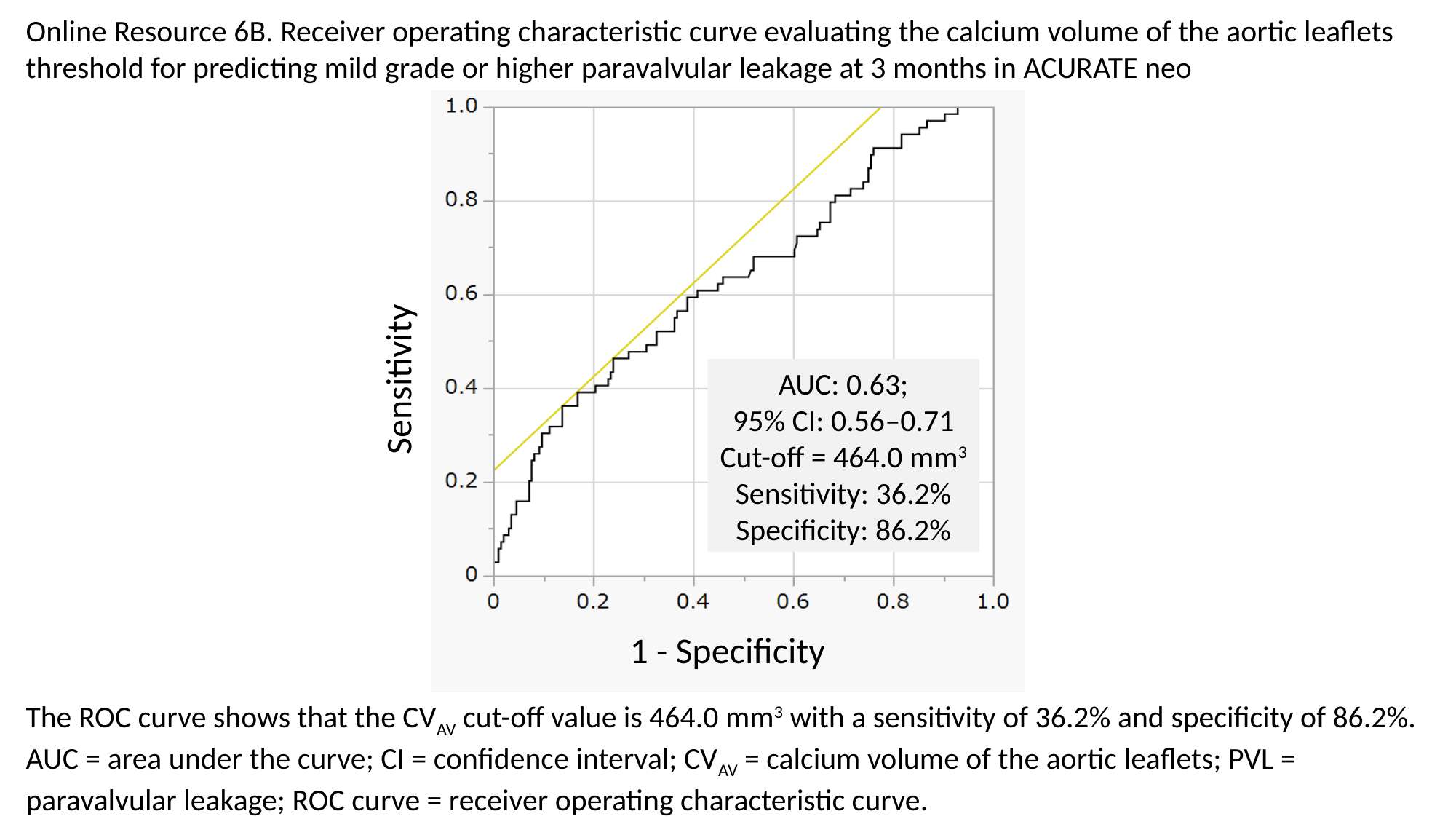

Online Resource 6B. Receiver operating characteristic curve evaluating the calcium volume of the aortic leaflets threshold for predicting mild grade or higher paravalvular leakage at 3 months in ACURATE neo
Sensitivity
AUC: 0.63;
95% CI: 0.56–0.71
Cut-off = 464.0 mm3
Sensitivity: 36.2%
Specificity: 86.2%
1 - Specificity
The ROC curve shows that the CVAV cut-off value is 464.0 mm3 with a sensitivity of 36.2% and specificity of 86.2%.
AUC = area under the curve; CI = confidence interval; CVAV = calcium volume of the aortic leaflets; PVL = paravalvular leakage; ROC curve = receiver operating characteristic curve.

## Slide 5
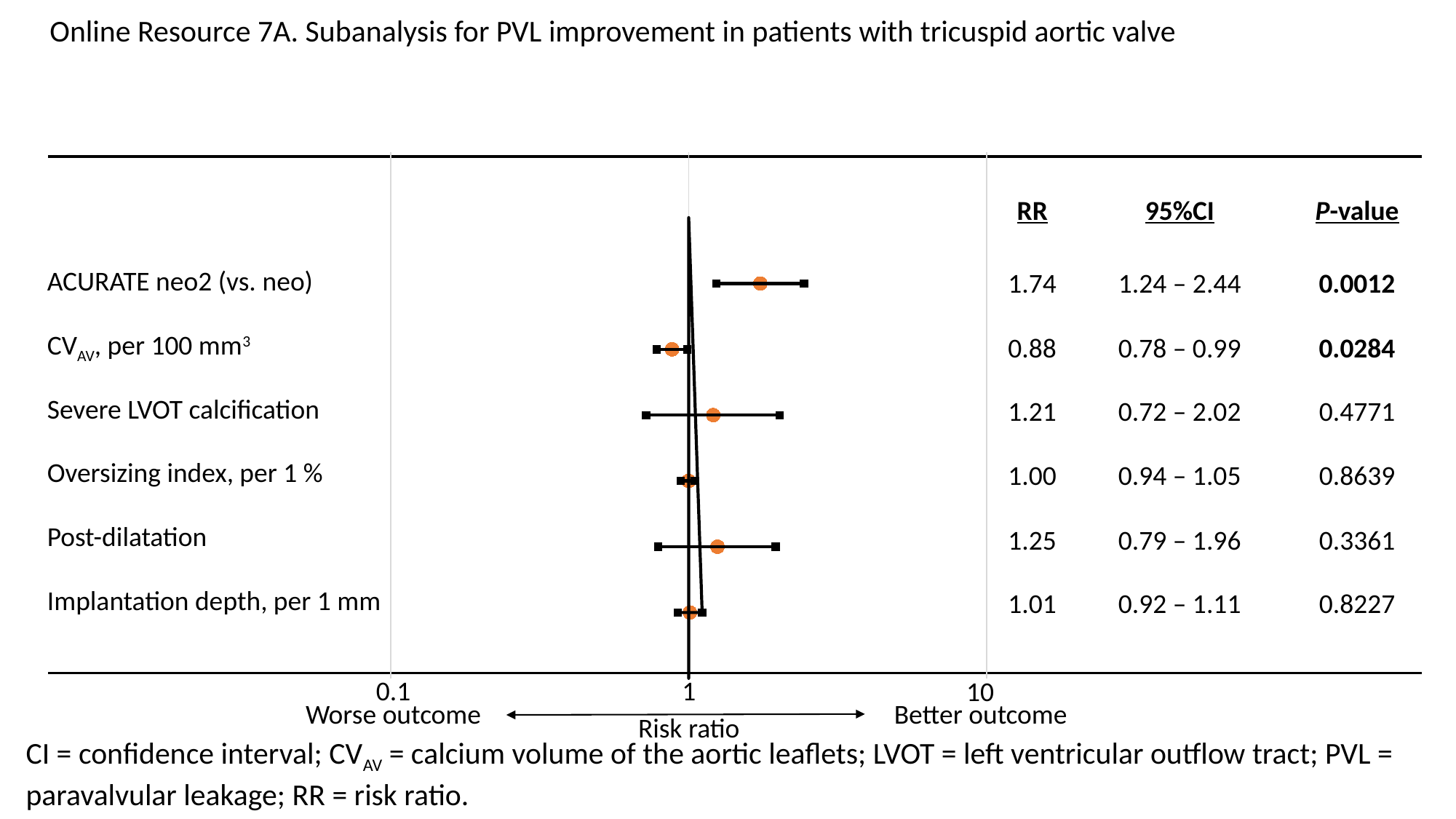

Online Resource 7A. Subanalysis for PVL improvement in patients with tricuspid aortic valve
### Chart
| Category | | |
|---|---|---|| RR | 95%CI | P-value |
| --- | --- | --- |
| 1.74 | 1.24 – 2.44 | 0.0012 |
| 0.88 | 0.78 – 0.99 | 0.0284 |
| 1.21 | 0.72 – 2.02 | 0.4771 |
| 1.00 | 0.94 – 1.05 | 0.8639 |
| 1.25 | 0.79 – 1.96 | 0.3361 |
| 1.01 | 0.92 – 1.11 | 0.8227 |
| |
| --- |
| ACURATE neo2 (vs. neo) |
| CVAV, per 100 mm3 |
| Severe LVOT calcification |
| Oversizing index, per 1 % |
| Post-dilatation |
| Implantation depth, per 1 mm |
1
0.1
10
Worse outcome
Better outcome
Risk ratio
CI = confidence interval; CVAV = calcium volume of the aortic leaflets; LVOT = left ventricular outflow tract; PVL = paravalvular leakage; RR = risk ratio.

## Slide 6
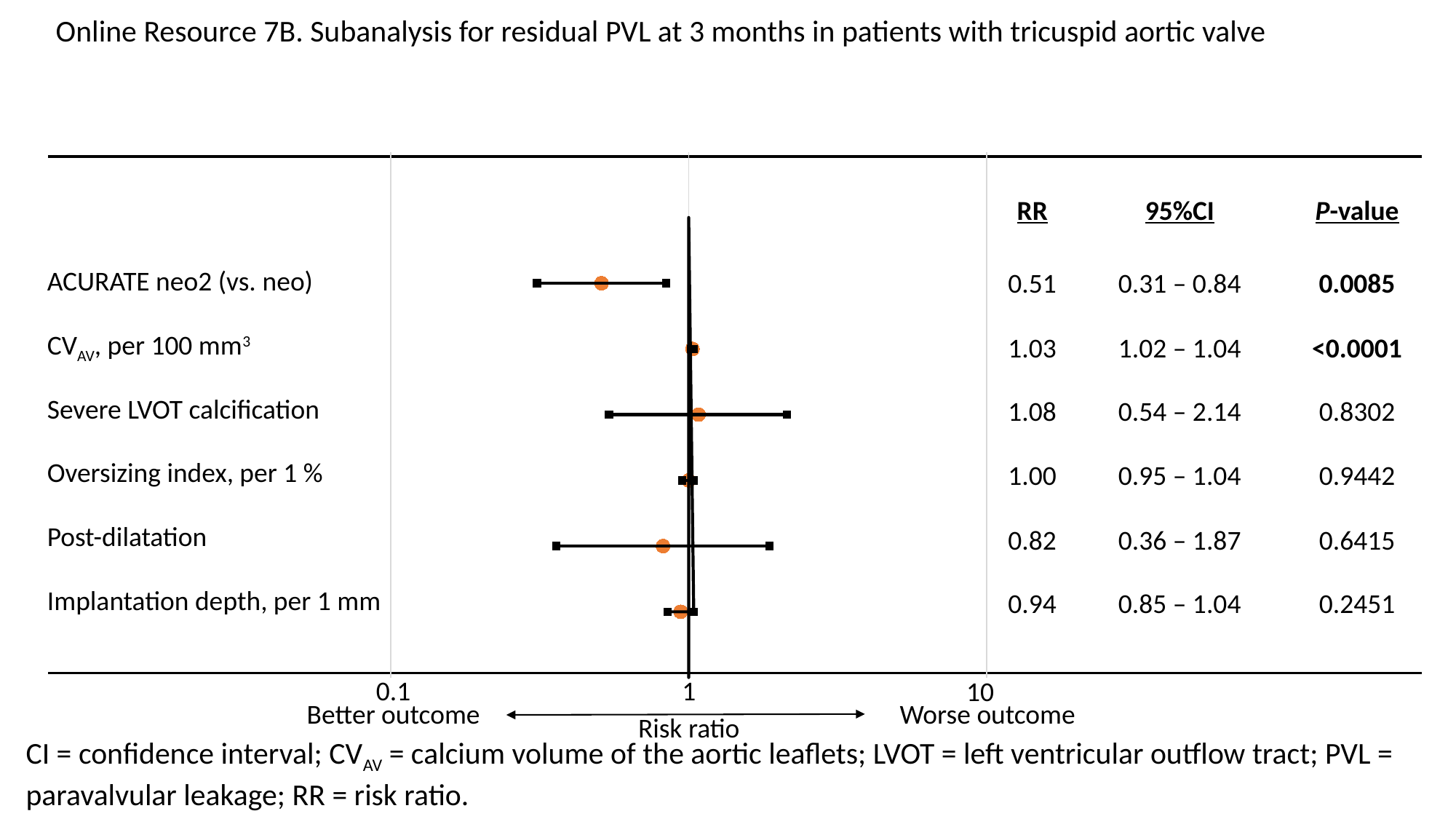

Online Resource 7B. Subanalysis for residual PVL at 3 months in patients with tricuspid aortic valve
### Chart
| Category | | |
|---|---|---|| RR | 95%CI | P-value |
| --- | --- | --- |
| 0.51 | 0.31 – 0.84 | 0.0085 |
| 1.03 | 1.02 – 1.04 | <0.0001 |
| 1.08 | 0.54 – 2.14 | 0.8302 |
| 1.00 | 0.95 – 1.04 | 0.9442 |
| 0.82 | 0.36 – 1.87 | 0.6415 |
| 0.94 | 0.85 – 1.04 | 0.2451 |
| |
| --- |
| ACURATE neo2 (vs. neo) |
| CVAV, per 100 mm3 |
| Severe LVOT calcification |
| Oversizing index, per 1 % |
| Post-dilatation |
| Implantation depth, per 1 mm |
1
0.1
10
Better outcome
Worse outcome
Risk ratio
CI = confidence interval; CVAV = calcium volume of the aortic leaflets; LVOT = left ventricular outflow tract; PVL = paravalvular leakage; RR = risk ratio.

## Slide 7
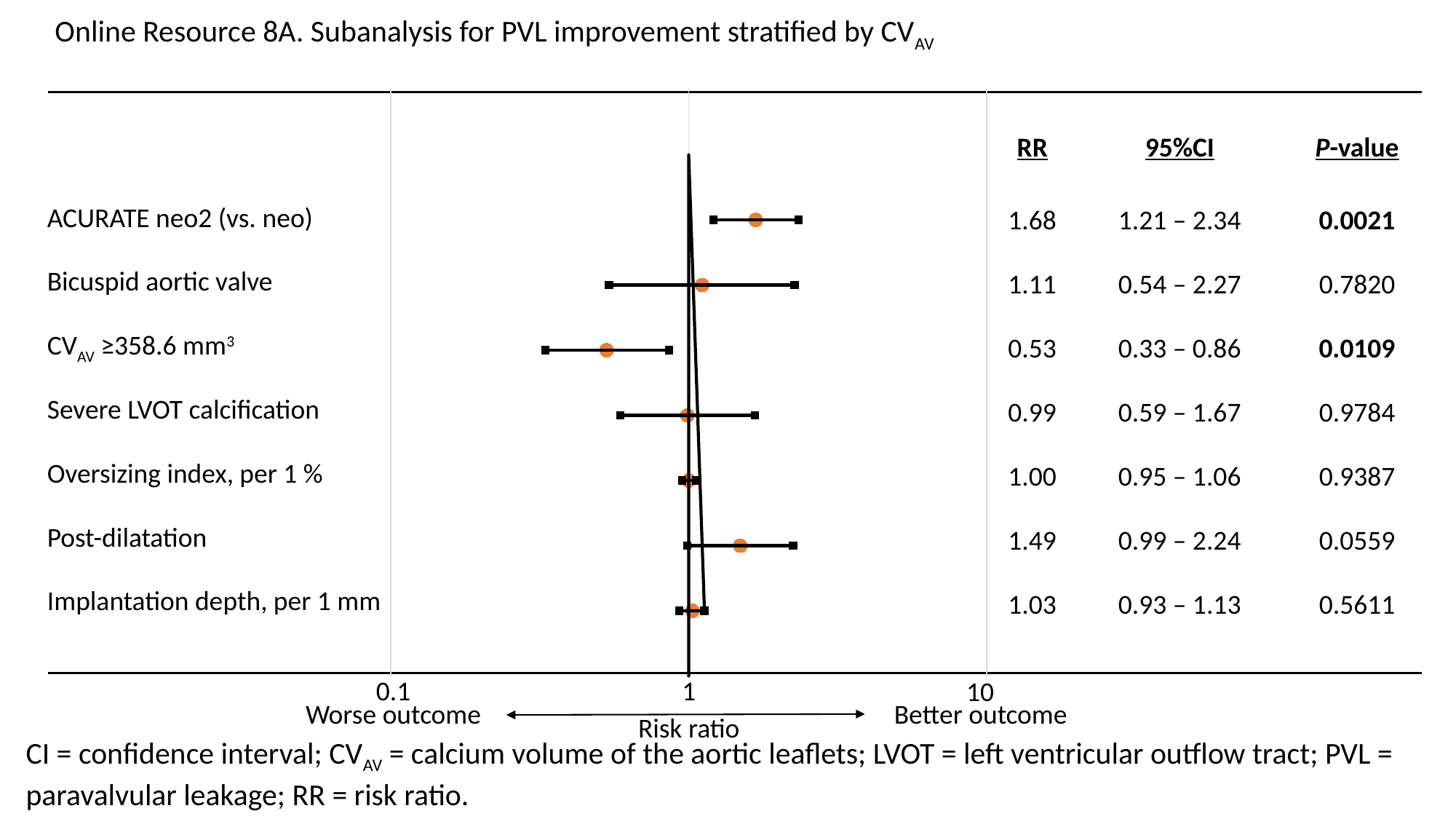

Online Resource 8A. Subanalysis for PVL improvement stratified by CVAV
### Chart
| Category | | |
|---|---|---|| RR | 95%CI | P-value |
| --- | --- | --- |
| 1.68 | 1.21 – 2.34 | 0.0021 |
| 1.11 | 0.54 – 2.27 | 0.7820 |
| 0.53 | 0.33 – 0.86 | 0.0109 |
| 0.99 | 0.59 – 1.67 | 0.9784 |
| 1.00 | 0.95 – 1.06 | 0.9387 |
| 1.49 | 0.99 – 2.24 | 0.0559 |
| 1.03 | 0.93 – 1.13 | 0.5611 |
| |
| --- |
| ACURATE neo2 (vs. neo) |
| Bicuspid aortic valve |
| CVAV ≥358.6 mm3 |
| Severe LVOT calcification |
| Oversizing index, per 1 % |
| Post-dilatation |
| Implantation depth, per 1 mm |
1
0.1
10
Worse outcome
Better outcome
Risk ratio
CI = confidence interval; CVAV = calcium volume of the aortic leaflets; LVOT = left ventricular outflow tract; PVL = paravalvular leakage; RR = risk ratio.

## Slide 8
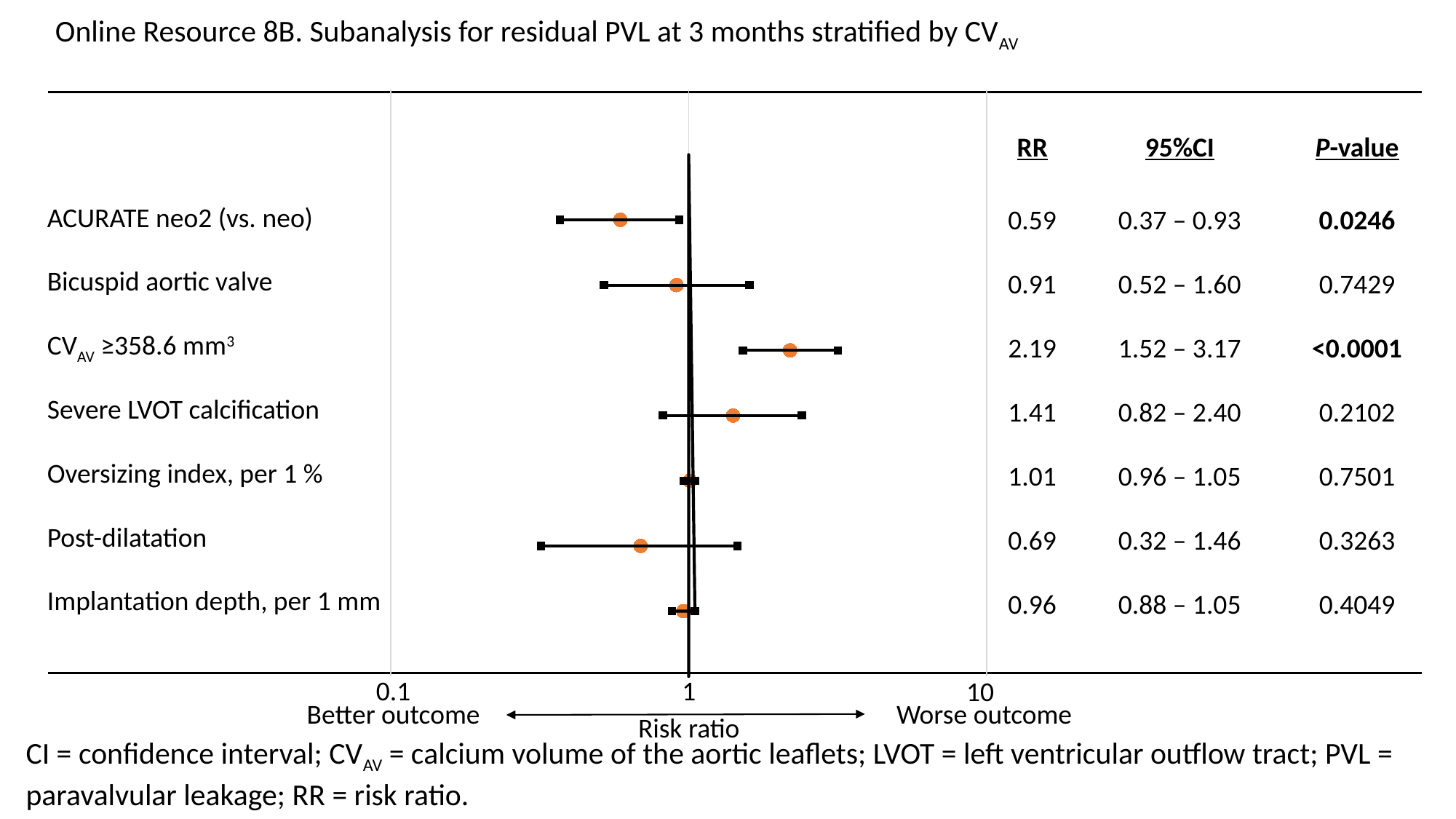

Online Resource 8B. Subanalysis for residual PVL at 3 months stratified by CVAV
### Chart
| Category | | |
|---|---|---|| RR | 95%CI | P-value |
| --- | --- | --- |
| 0.59 | 0.37 – 0.93 | 0.0246 |
| 0.91 | 0.52 – 1.60 | 0.7429 |
| 2.19 | 1.52 – 3.17 | <0.0001 |
| 1.41 | 0.82 – 2.40 | 0.2102 |
| 1.01 | 0.96 – 1.05 | 0.7501 |
| 0.69 | 0.32 – 1.46 | 0.3263 |
| 0.96 | 0.88 – 1.05 | 0.4049 |
| |
| --- |
| ACURATE neo2 (vs. neo) |
| Bicuspid aortic valve |
| CVAV ≥358.6 mm3 |
| Severe LVOT calcification |
| Oversizing index, per 1 % |
| Post-dilatation |
| Implantation depth, per 1 mm |
1
0.1
10
Better outcome
Worse outcome
Risk ratio
CI = confidence interval; CVAV = calcium volume of the aortic leaflets; LVOT = left ventricular outflow tract; PVL = paravalvular leakage; RR = risk ratio.

## Slide 9
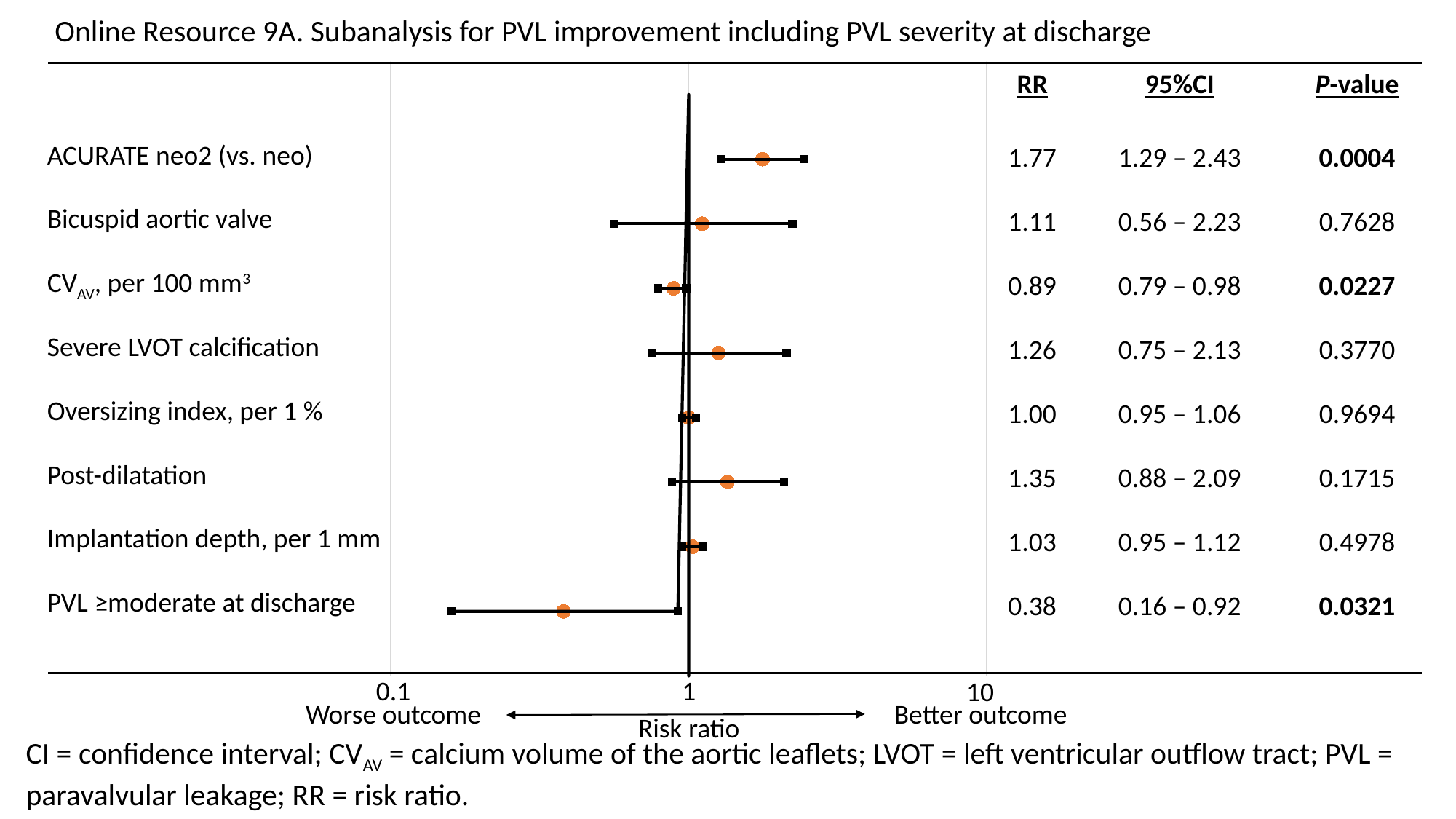

Online Resource 9A. Subanalysis for PVL improvement including PVL severity at discharge
### Chart
| Category | | |
|---|---|---|| RR | 95%CI | P-value |
| --- | --- | --- |
| 1.77 | 1.29 – 2.43 | 0.0004 |
| 1.11 | 0.56 – 2.23 | 0.7628 |
| 0.89 | 0.79 – 0.98 | 0.0227 |
| 1.26 | 0.75 – 2.13 | 0.3770 |
| 1.00 | 0.95 – 1.06 | 0.9694 |
| 1.35 | 0.88 – 2.09 | 0.1715 |
| 1.03 | 0.95 – 1.12 | 0.4978 |
| 0.38 | 0.16 – 0.92 | 0.0321 |
| |
| --- |
| ACURATE neo2 (vs. neo) |
| Bicuspid aortic valve |
| CVAV, per 100 mm3 |
| Severe LVOT calcification |
| Oversizing index, per 1 % |
| Post-dilatation |
| Implantation depth, per 1 mm |
| PVL ≥moderate at discharge |
1
0.1
10
Worse outcome
Better outcome
Risk ratio
CI = confidence interval; CVAV = calcium volume of the aortic leaflets; LVOT = left ventricular outflow tract; PVL = paravalvular leakage; RR = risk ratio.

## Slide 10
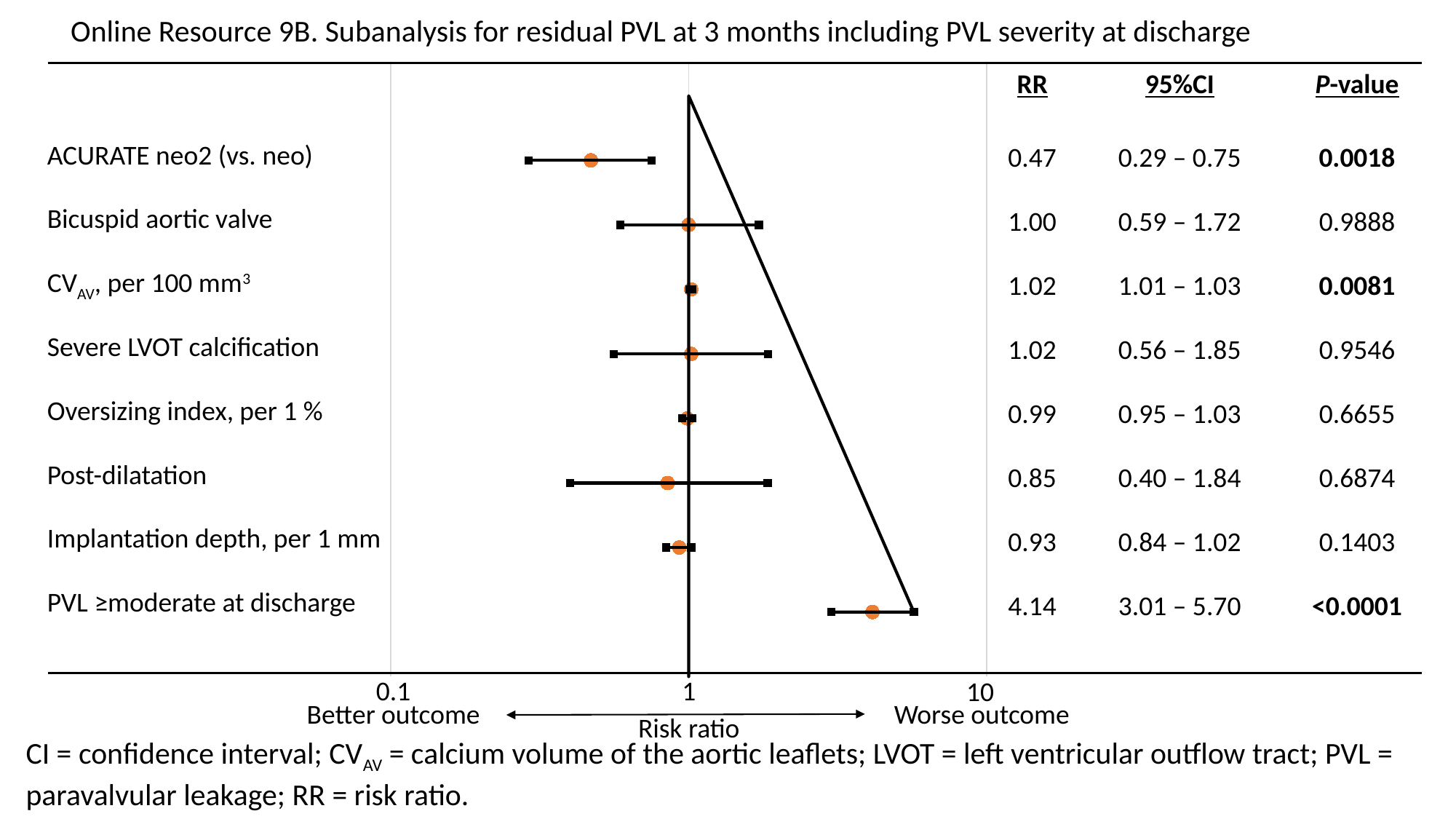

Online Resource 9B. Subanalysis for residual PVL at 3 months including PVL severity at discharge
### Chart
| Category | | |
|---|---|---|| RR | 95%CI | P-value |
| --- | --- | --- |
| 0.47 | 0.29 – 0.75 | 0.0018 |
| 1.00 | 0.59 – 1.72 | 0.9888 |
| 1.02 | 1.01 – 1.03 | 0.0081 |
| 1.02 | 0.56 – 1.85 | 0.9546 |
| 0.99 | 0.95 – 1.03 | 0.6655 |
| 0.85 | 0.40 – 1.84 | 0.6874 |
| 0.93 | 0.84 – 1.02 | 0.1403 |
| 4.14 | 3.01 – 5.70 | <0.0001 |
| |
| --- |
| ACURATE neo2 (vs. neo) |
| Bicuspid aortic valve |
| CVAV, per 100 mm3 |
| Severe LVOT calcification |
| Oversizing index, per 1 % |
| Post-dilatation |
| Implantation depth, per 1 mm |
| PVL ≥moderate at discharge |
1
0.1
10
Better outcome
Worse outcome
Risk ratio
CI = confidence interval; CVAV = calcium volume of the aortic leaflets; LVOT = left ventricular outflow tract; PVL = paravalvular leakage; RR = risk ratio.

## Slide 11
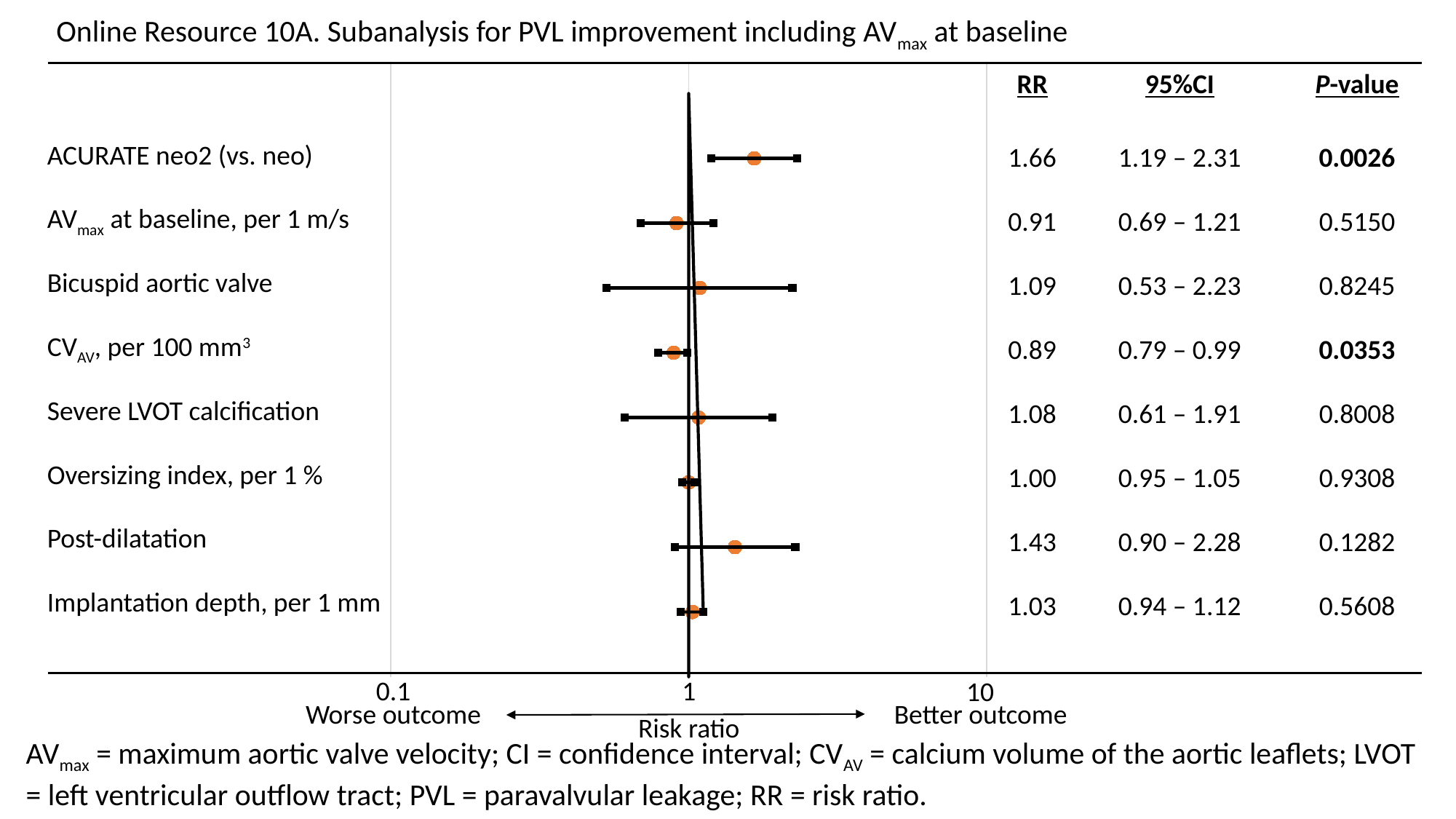

Online Resource 10A. Subanalysis for PVL improvement including AVmax at baseline
### Chart
| Category | | |
|---|---|---|
| RR | 95%CI | P-value |
| --- | --- | --- |
| 1.66 | 1.19 – 2.31 | 0.0026 |
| 0.91 | 0.69 – 1.21 | 0.5150 |
| 1.09 | 0.53 – 2.23 | 0.8245 |
| 0.89 | 0.79 – 0.99 | 0.0353 |
| 1.08 | 0.61 – 1.91 | 0.8008 |
| 1.00 | 0.95 – 1.05 | 0.9308 |
| 1.43 | 0.90 – 2.28 | 0.1282 |
| 1.03 | 0.94 – 1.12 | 0.5608 |
| |
| --- |
| ACURATE neo2 (vs. neo) |
| AVmax at baseline, per 1 m/s |
| Bicuspid aortic valve |
| CVAV, per 100 mm3 |
| Severe LVOT calcification |
| Oversizing index, per 1 % |
| Post-dilatation |
| Implantation depth, per 1 mm |
1
0.1
10
Worse outcome
Better outcome
Risk ratio
AVmax = maximum aortic valve velocity; CI = confidence interval; CVAV = calcium volume of the aortic leaflets; LVOT = left ventricular outflow tract; PVL = paravalvular leakage; RR = risk ratio.

## Slide 12
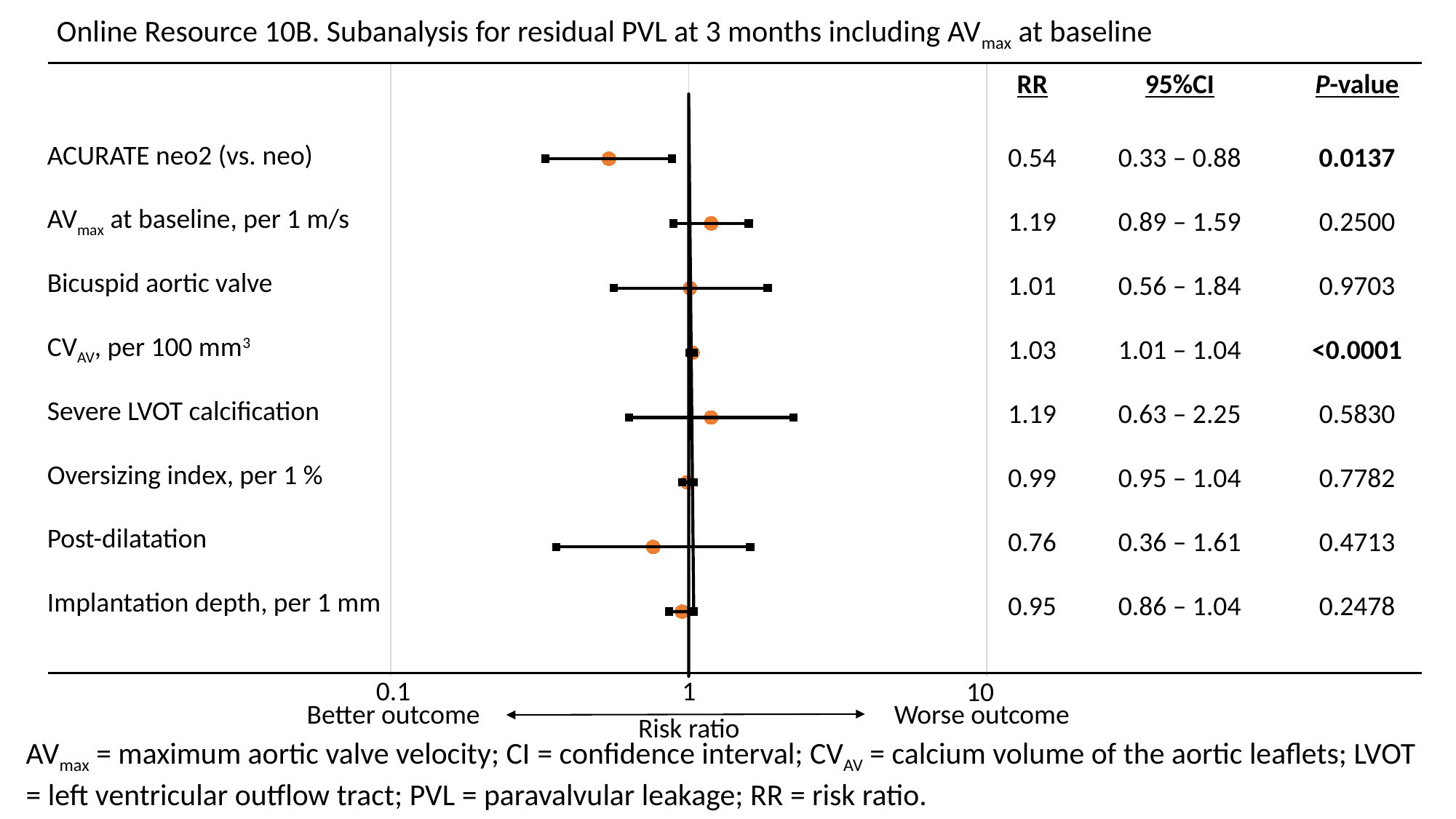

Online Resource 10B. Subanalysis for residual PVL at 3 months including AVmax at baseline
### Chart
| Category | | |
|---|---|---|| RR | 95%CI | P-value |
| --- | --- | --- |
| 0.54 | 0.33 – 0.88 | 0.0137 |
| 1.19 | 0.89 – 1.59 | 0.2500 |
| 1.01 | 0.56 – 1.84 | 0.9703 |
| 1.03 | 1.01 – 1.04 | <0.0001 |
| 1.19 | 0.63 – 2.25 | 0.5830 |
| 0.99 | 0.95 – 1.04 | 0.7782 |
| 0.76 | 0.36 – 1.61 | 0.4713 |
| 0.95 | 0.86 – 1.04 | 0.2478 |
| |
| --- |
| ACURATE neo2 (vs. neo) |
| AVmax at baseline, per 1 m/s |
| Bicuspid aortic valve |
| CVAV, per 100 mm3 |
| Severe LVOT calcification |
| Oversizing index, per 1 % |
| Post-dilatation |
| Implantation depth, per 1 mm |
1
0.1
10
Better outcome
Worse outcome
Risk ratio
AVmax = maximum aortic valve velocity; CI = confidence interval; CVAV = calcium volume of the aortic leaflets; LVOT = left ventricular outflow tract; PVL = paravalvular leakage; RR = risk ratio.

## Slide 13
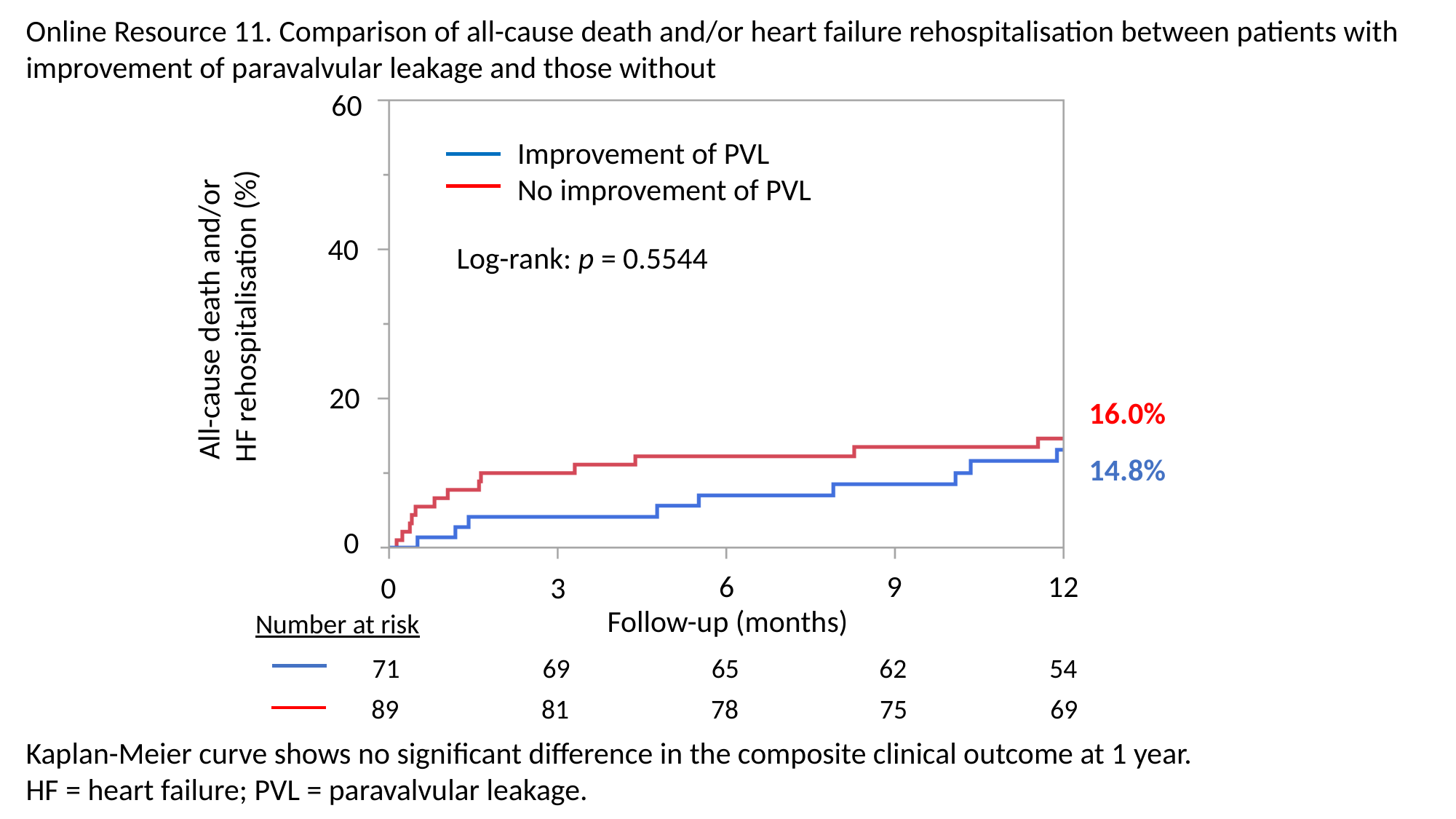

Online Resource 11. Comparison of all-cause death and/or heart failure rehospitalisation between patients with improvement of paravalvular leakage and those without
60
Improvement of PVL
No improvement of PVL
40
Log-rank: p = 0.5544
All-cause death and/or
HF rehospitalisation (%)
20
16.0%
14.8%
0
6
9
12
0
3
Follow-up (months)
Number at risk
71
69
62
54
65
78
81
89
75
69
Kaplan-Meier curve shows no significant difference in the composite clinical outcome at 1 year.
HF = heart failure; PVL = paravalvular leakage.
